# Association between BK polyomavirus and prostate cancer: a systematic review and meta-analysis

**DOI:** 10.3389/fonc.2026.1764752

**Published:** 2026-05-08

**Authors:** Bingyu Xiang, Shuaibin Wang, Xinrong Zhang, Jiaxin Zhao, Mingxia Zhang, Juan He

**Affiliations:** 1 School of Basic Medicine, Shanxi Medical University, Taiyuan, China; 2 Academy of Medical Sciences, Shanxi Medical University, Taiyuan, China; 3 Third Hospital of Shanxi Medical University, Shanxi Bethune Hospital, Shanxi Academy of Medical Sciences, Tongji Shanxi Hospital, Taiyuan, China; 4 Laboratory Department, Shanxi Children’s Hospital, Taiyuan, China; 5 Department of Pathology, Shanxi Bethune Hospital, Shanxi Academy of Medical Sciences, Third Hospital of Shanxi Medical University, Tongji Shanxi Hospital, Taiyuan, China

**Keywords:** BK polyomavirus, meta-analysis, prostate cancer, risk factor, systematic review

## Abstract

Prostate cancer (PCa) is the second most commonly diagnosed malignancy in men worldwide, but the role of viral infection, particularly BK polyomavirus (BKPyV), remains controversial. To clarify this association, we conducted a systematic review and meta-analysis of 23 studies published between January 1, 2000, and May 1, 2025. The pooled prevalence of BKPyV in PCa tissues was 22% (95% CI: 12–34%; I²= 96.27%), indicating that the inter-study heterogeneity is high. Eleven case-control studies showed that BKPyV infection was associated with increased odds of PCa (pooled OR = 3.04, 95% CI: 2.15–4.30; I²= 31.7%). Subgroup analyses by specimen type, detection method, molecular target, study location, and Gleason score demonstrated a consistent positive association, although residual heterogeneity persisted. Because all included studies relied on PCR-based detection, the identified viral DNA cannot distinguish episomal, integrated, defective, or transcriptionally active states, nor establish temporal order or causality. Overall, BKPyV may represent a plausible cofactor or context-dependent marker in PCa, and the observed pattern is compatible with several hypotheses, including hit-and-run, microenvironment driven phenomenon, and a mixed phenomenon. Standardized molecular assays and more complete clinicopathological annotation are needed in future studies.

**Systematic Review Registration:**

https://www.crd.york.ac.uk/PROSPERO/view/CRD420251179044, identifier CRD420251179044.

## 1 Introduction

Prostate cancer (PCa) is a major global public health issue and one of the leading causes of cancer-related death in men (1). According to recent epidemiological data, PCa is the second most commonly diagnosed cancer in men in the world and the fifth leading cause of cancer death. In 2022, there were an estimated 1,466,680 new cases and 396,792 deaths (2). Current treatment strategies for PCa are individualized according to tumor stage and risk stratification (3). For localized low−risk disease, active surveillance is widely used in clinical practice to avoid overtreatment. For intermediate−risk and high−risk tumors, radical prostatectomy and/or radiotherapy are generally applied, often in combination with androgen deprivation therapy (ADT). For advanced or metastatic disease, systemic therapies based on ADT remain the cornerstone of treatment, often augmented with androgen receptor pathway inhibitors, chemotherapeutic agents, radiopharmaceuticals, immunotherapy, or molecular targeted drugs (4, 5). Despite the continuous progress of treatment methods, a considerable proportion of patients still have drug resistance or worsening of the disease, underscoring the need to clarify carcinogenic mechanisms and identify potentially modifiable risk factors (4). PCa is a multifactorial disease influenced by many factors. Established risk factors include older age, Black race/ethnicity, and a family history of the disease (6). However, other potential risk factors, such as obesity, dietary habits, and fitness, are still being investigated for their contributions to PCa risk (7–9).

Viral infection is another important risk factor. It is estimated that about 15 to 20% of all human cancer cases in the world are caused by viral infection (10, 11). In recent years, more and more studies have reported the association between PCa and specific viral infections, including human papillomavirus, cytomegalovirus, human herpes simplex virus type 2, Epstein-Barr virus, and polyomaviruses (12). Therefore, the potential link between viral infection and PCa has attracted increasing attention.

BK polyomavirus (BKPyV) is a small, non-enveloped virus with a double-stranded DNA genome and belongs to the Polyomaviridae family (13). Primary infection typically occurs asymptomatically during early childhood and is ubiquitous worldwide, with adult seroprevalence estimated at 80–90% (14). BKPyV also exhibits geographic genetic heterogeneity, with the distribution of viral genotypes and subtypes varying across populations and regions (15). Following the initial exposure, the virus exhibits a strong tissue tropism for the human urogenital tract. It establishes a life-long latent infection primarily targeting the renal tubular epithelial cells, the urothelium of the bladder, and the prostate (16). BKPyV maintains this widespread latent infection in healthy individuals but exhibits a propensity for reactivation under conditions of cellular immune dysfunction, and is frequently observed in renal transplant recipients (17, 18).

The BKPyV genome comprises both early and late transcription units that govern its oncogenicity. The late region produces viral structural components (VP1, VP2, and VP3) and the agnoprotein. In contrast, the primary oncogenic components, namely the Large T antigen (TAg) and small t antigen, are derived from the early transcription region (19). As the main viral oncoprotein, TAg promotes cell cycle disorder by synergistic destruction of retinoblastoma protein (pRb) and p53 pathways. By binding pocket proteins of the retinoblastoma family, TAg releases E2F transcription factors and drives premature G1/S transition, thereby facilitating unscheduled S phase entry and proliferative signaling (20, 21). Concurrently, TAg binds p53 and dampens p53-dependent DNA-damage responses, enabling the survival and expansion of genomically unstable cells (20). In addition, the small t antigen acts synergistically with TAg by interacting with host protein phosphatase 2A (PP2A), inhibiting its activity to further stimulate oncogenic signaling pathways necessary for cellular transformation (21). More recent mechanistic studies suggest that BKPyV-associated oncogenicity may extend beyond these canonical pathways. Experimental studies show that BKPyV can activate the DNA damage response mechanism and maintain a state similar to the S phase to support viral replication, while single-cell analyses indicate that robust TAg expression and productive replication depend on an initial host S phase and subsequent host re-replication (22–24). In differentiated urothelial models, BKPyV infection has also been shown to induce APOBEC3-associated genomic damage, which provides another mechanism for the instantaneous viral activity to leave mutative changes in host cells (23). In summary, these findings expand the mechanism of BKPyV’s possible involvement in carcinogenicity. Consequently, both *in vitro* and *in vivo* animal studies have provided robust evidence of BKPyV’s oncogenic potential through these coordinated molecular events (25, 26) and BKPyV has been classified by the World Health Organization as “possibly carcinogenic to humans” (27, 31).

Given that the prostate gland is part of the urogenital tract and a common site for BKPyV latent infection and viral shedding, researchers have recently begun to investigate a potential link between BKPyV and the pathogenesis of PCa. Tavassoli et al. used a highly sensitive nested PCR assay targeting the TAg region in formalin-fixed paraffin-embedded (FFPE) prostate specimens and reported a markedly higher prevalence of BKPyV DNA in PCa tissues than in benign prostatic hyperplasia (BPH) tissues, suggesting a positive association between intraprostatic BKPyV infection and cancer risk (27). In contrast, Malekshahi et al., who examined FFPE tissues from PCa and BPH patients with conventional PCR for the TAg region, did not detect BKPyV DNA in any specimen (28). Similarly, Bergh et al. applied nested PCR directed against the VP1 in prostate biopsies from cancer patients and non-cancer controls and also failed to demonstrate BKPyV in either group (29). From the methodological level, factors such as sample type differences, DNA quality in FFPE tissue processing, primer/probe design and analytical sensitivity of detection methods may lead to false negative or false positive results in the study. From a biological perspective, the oncogenic potential of BKPyV mainly comes from its early region, particularly TAg, which interferes with the pRb-E2F and p53 pathways; and the VP1 gene, as a late structural gene, is usually expressed in the productive replication of the virus, rather than in the latent or termination of infection (20, 21). Taken together, these considerations highlight substantial between-study heterogeneity driven by both biological and technical factors.

In summary, the existing literature reports contradictory results, and the association between BKPyV and PCa is not clear. Some of these conflicting findings may reflect underdiagnosis of BKPyV in prostate tissue, particularly when viral DNA is present at low levels or unevenly distributed, thereby limiting further research into the molecular determinants of BKPyV in this disease context. Therefore, this systematic review and meta-analysis aims to comprehensively evaluate the prevalence of BKPyV in PCa tissues and its potential association with PCa risk.

## 2 Methods

The planning and implementation of this study followed the Preferred Reporting Items for Systematic Reviews and Meta-Analyses (PRISMA) statement (30). The protocol was registered in PROSPERO (CRD420251179044). To establish our study selection criteria, we utilized the PECO (Population, Exposure, Comparison, and Outcome) framework, focusing on patients with prostate cancer (P), BKPyV infection (E), individuals without prostate cancer, or non-cancerous prostate tissue specimens (applicable to case-control studies) (C), and the prevalence of BKPyV as well as its risk association with prostate cancer (O). Based on this framework, the main objectives of this study are divided into two parts: first, to determine the prevalence of BKPyV in PCa tissues; second, to investigate the association between BKPyV infection and PCa risk.

### 2.1 Literature search strategy

Electronic databases, including PubMed, Web of Science, ScienceDirect, ProQuest, Scopus and Embase, were systematically searched for relevant studies investigating the prevalence and role of BKPyV in PCa. The retrieval time range of the main database is from January 1, 2000 to May 1, 2025. This start date was selected *a priori* to improve methodological comparability across studies, because earlier tissue-based BKPyV detection studies were more likely to use less standardized molecular assays with substantial variation in primer design, target region selection, analytical sensitivity, and laboratory protocols. Key search terms included Medical Subject Headings (MeSH) terms such as “Prostatic Neoplasms,” “BK Virus,” and “Polyomavirus,” along with their synonyms. These terms were combined using the Boolean operators “OR” and “AND” in the title/abstract/keywords fields (**Supplementary Table S1**). We also conducted a “snowballing” search to identify more studies by searching for the list of references of publications that meet the full text review conditions and using Google Scholar to identify and screen the studies that refer to them.

Literature retrieval and study selection were independently conducted by two reviewers (Bingyu Xiang and Shuaibin Wang). Eligibility decisions were made according to the prespecified inclusion and exclusion criteria established using the PECO framework and the registered study protocol. Any disagreements were resolved through discussion between the two reviewers, and final inclusion was determined by consensus based on the predefined inclusion and exclusion criteria. Studies judged to be of low methodological quality were excluded according to the predefined NOS thresholds (0–3 for case-control studies and 0–4 for cross-sectional studies).

### 2.2 Inclusion criteria

1. Studies that assessed the association between PCa and BKPyV infection, specifically those utilizing cross-sectional or case-control studies.
2. Studies that utilized various types of Polymerase chain reaction (PCR) techniques to examine the presence of BKPyV in PCa tissue.
3. Studies that reported the prevalence of BKPyV in PCa patients or provided one of three measures of association between BKPyV in PCa tissue and control groups: Odds Ratio (OR), Relative Risk (RR), or Prevalence Rate Ratio (PRR). Studies from which an OR could be directly calculated from the raw data were also included.
4. The full-text original articles published from January 1, 2000, and May 1, 2025.

### 2.3 Exclusion criteria

1. Studies judged to be of low quality after formal assessment were excluded. Specifically, studies scoring 0–3 on the Newcastle-Ottawa Scale (NOS) for case-control studies and 0–4 on the modified NOS for cross-sectional studies were classified as low quality.
2. Reviews, case reports, commentaries, conference abstracts, cell line studies, and any other publication types without relevant extractable data.
3. Studies that assessed BKPyV presence using serological assays or other non-PCR tissue-based methods, such as *in situ* hybridization (ISH) and immunohistochemistry (IHC), were excluded. Serology reflects past exposure and cannot confirm intratumoral infection (31). By contrast, ISH and IHC provide different biological readouts, including tissue localization and protein expression, and their positivity definitions are not directly comparable with PCR-based positivity estimates for pooled prevalence or case-control effect analysis. We acknowledge that ISH/IHC-based studies may provide complementary mechanistic information, but these endpoints were outside the scope of the present quantitative meta-analysis.
4. Studies that utilized non-cancerous tissue samples or substitute specimens (e.g., plasma or urine) instead of PCa tissue.
5. Studies conducted exclusively in immunocompromised populations (e.g., HIV/AIDS patients or transplant recipients) were excluded because impaired host immune surveillance may substantially alter BKPyV reactivation dynamics, viral burden, and tissue positivity, such that the resulting prevalence estimates and association measures may not be directly comparable with those from the general prostate cancer population.

### 2.4 Data extraction

Data extraction was performed independently by two researchers (Bingyu Xiang and Xinrong Zhang) from the included studies. Extracted information included: first author name, study design, publication year, study population characteristics (study location, sample size, etc.), specimen type, methods used to assess BKPyV presence, molecular target region, the number of positive and negative cases for BKPyV in cancerous or non-cancerous tissues, and Gleason score. All data were double-entered into standardized data extraction forms to minimize data entry errors.

### 2.5 Quality assessment

Two investigators (Bingyu Xiang and Shuaibin Wang) independently assessed the quality of the included studies using the NOS or its modified version for cross-sectional studies. The studies included in this meta-analysis were observational studies, including case-control studies and cross-sectional studies. The NOS was used to evaluate the quality of all included case-control studies. The scale comprises three domains: Selection, Comparability, and Exposure. The scoring criteria for the NOS were as follows: a total score of 0–3 points indicates low quality, 4–6 points indicates medium quality, and 7–9 points indicates high quality.

Similarly, the quality of all included cross-sectional studies was evaluated using a modified version of the NOS for cross-sectional studies (32) (**Supplementary Table S2**). The tool was modified for the purpose of this study. This modified tool is also structured into three domains: Selection, Comparability, and Outcome. The scoring criteria for this scale were as follows: a total score of 0–4 points indicates low quality, 5–7 points indicates medium quality, and 8–10 points indicates high quality.

Studies classified as low quality according to these predefined thresholds were excluded before meta-analysis.

### 2.6 Statistical analysis

The metaprop command was used to pool the overall prevalence of BKPyV in PCa tissues. This study adopted a random-effect meta-analysis framework, and conducted subgroup analyses according to specimen type, study location, detection method, molecular target region of the PCR and Gleason score. Heterogeneity was assessed using Cochran’s Q test and the I² statistic. The Cochran’s Q test examines whether observed between-study differences exceed those expected from sampling error alone, and I² describes the percentage of total variability across studies that is due to true heterogeneity rather than chance. Formal publication bias assessment was not performed for the prevalence meta-analysis because funnel plots and Egger’s/Begg’s tests were developed for comparative data and depend on assumptions about “positive” versus “negative” study findings that are not well defined in prevalence meta-analysis (33, 34). For case-control studies, a meta-analysis was performed on the risk estimates of PCa associated with BKPyV infection exposure. ORs were calculated from 2×2 tables, with a continuity correction of 0.5 applied when zero cells occurred. Pooled ORs with 95% confidence intervals (CIs) were estimated using a fixed-effect inverse-variance model, and heterogeneity was quantified by Q and I². We stratified the analyses by specimen type, study location, detection method, molecular target region of the PCR and Gleason score to assess the impact of grouping factors on the results and identify the origins of heterogeneity. The Egger’s test and the funnel plots were used to evaluate the publication bias included in the study. The Trim and Fill method was applied to estimate the potential impact of publication bias. In addition, 95% prediction intervals (PIs) were calculated for the main pooled estimates, and sensitivity analyses were carried out using the leave-one-out method to assess the impact of each individual study on the estimation of both the overall pooled prevalence and the pooled OR.

For Gleason score, our aim was to determine whether BKPyV detection and its association with PCa vary by tumor aggressiveness. Gleason score categories were defined as 6, 7, and 8–10. Because several studies reported multiple Gleason strata from the same case series and, in case-control studies, used a shared control group, stratum-specific prevalence and OR estimates within a study are correlated and should not be treated as independent studies. Therefore, beyond reporting grade-specific pooled prevalence and ORs, we performed within-study comparisons across Gleason categories. Prevalence ratios (PRs) were used to compare BKPyV detection across Gleason strata, and ratios of odds ratios (RORs) were used to compare the strength of the BKPyV and PCa association across strata. These contrasts were pooled on the log scale, which preserves study independence and avoids double-counting shared controls.

All statistical tests were two-tailed tests. All analyses were set at a significance level of *p* < 0.05, considered statistically significant, except for the heterogeneity test, which used a threshold of *p* < 0.10. Most of the analysis was completed using Stata 18.0 (StataCorp, College Station, TX, USA). PIs were calculated by R version 4.5.2 (The R Foundation, Vienna, Austria).

## 3 Results

### 3.1 Literature search results

**Figure 1:**
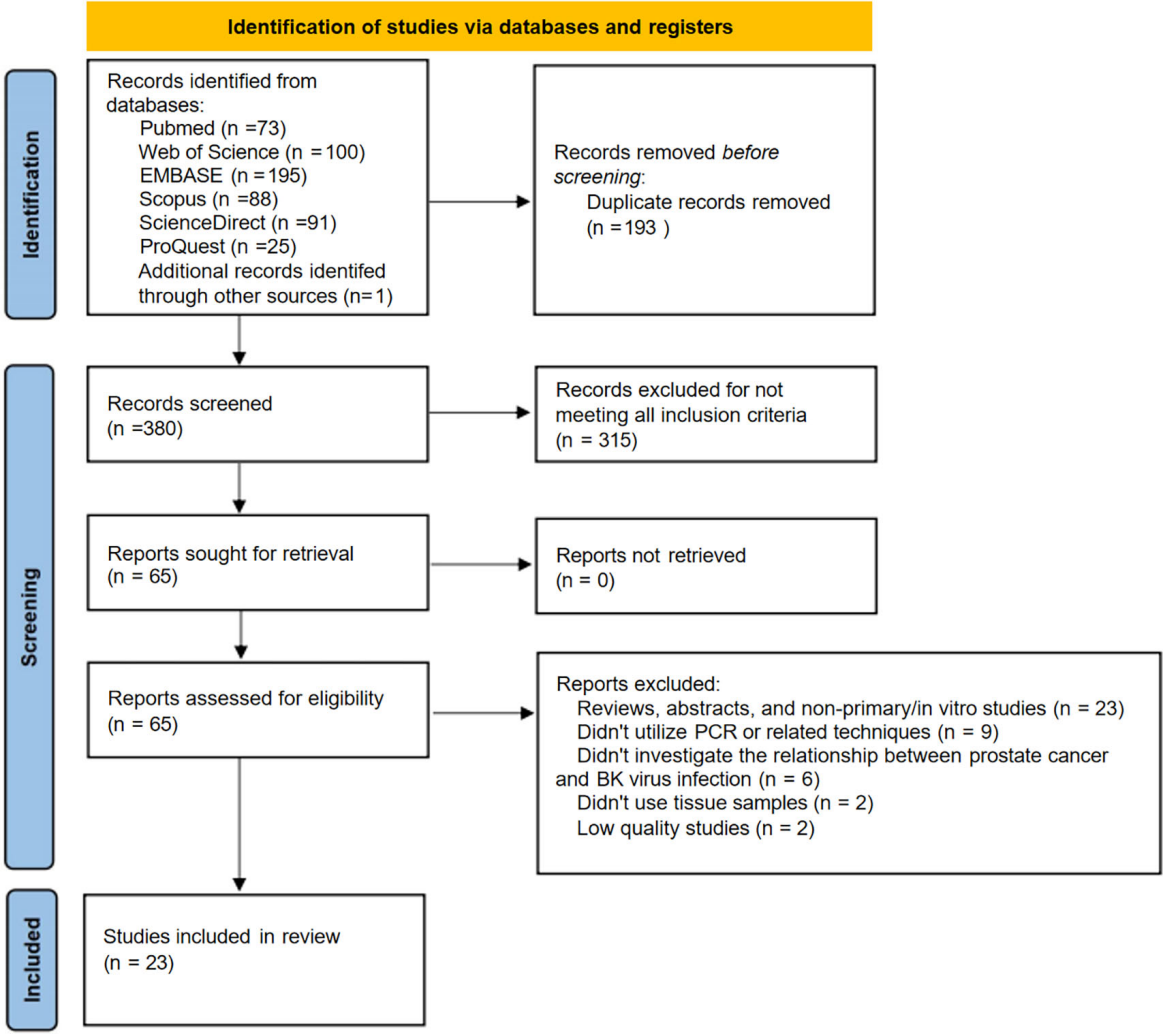
Flowchart depicting the literature search and selection process according to PRISMA guidelines.

According to the pre-set search strategy, a total of 573 records were obtained in the initial database retrieval. After deleting 193 duplicate records, 380 unique articles were screened out by reviewing titles and abstracts, and 315 unrelated studies were finally excluded. The eligibility of the remaining 65 articles was then assessed through full-text review. During the full-text review stage, we identified 42 studies that required exclusion for the following reasons: 23 were review articles, pathology reports, commentaries, conference abstracts, or cell line studies; 9 did not utilize PCR or related techniques; 6 were excluded due to inappropriate study design or irrelevant topics (e.g., *in vitro* models, therapeutic studies, or non-prostate malignancies); 2 did not use tissue samples; and 2 were excluded due to low methodological quality. Finally, 23 articles met the eligibility requirements and were retained for the meta-analysis (**Figure 1**). The characteristics were demonstrated in **Table 1**.

**Table 1:** Characteristics of studies included in the systematic review and meta-analysis.

First author	Year	Country	Study type	Detection method	Sample type	PCR target region	PCa cases (pos/total)	Controls (pos/total)
Tavassoli (27)	2023	Iran	Case-control	Nested-PCR	FFPE tissue	TAg	26/49	7/49
Shen (19)	2021	China	Case-control	Nested-PCR	FFPE tissue	TAg	3/76	0/30
Malekshahi (28)	2020	Iran	Case-control	PCR	FFPE tissue	TAg	0/64	0/57
Gorish (35)	2019	Sudan	Case-control	PCR	FFPE tissue	TAg	16/17	2/4
Taghavi (36)	2015	Iran	Case-control	PCR	FFPE tissue	VP1	17/60	9/60
Zhong (37)	2013	China	Case-control	Nested-PCR and PCR	Fresh tissue	TAg and VP1	6/32	1/50
Delbue (38)	2013	Italy	Case-control	qPCR	Fresh tissue	VP1	55/328	26/385
Sais (39)	2012	Switzerland	Case-control	qPCR	FFPE tissue	TAg	18/43	12/38
Martinez-Fierro (40)	2010	Mexico	Case-control	Nested-PCR	Fresh tissue	TAg	0/55	0/75
Russo (41)	2008	Italy	Case-control	qPCR	Fresh tissue	TAg	22/26	0/12
Bergh (29)	2007	Sweden	Case-control	Nested-PCR	FFPE tissue	VP1	0/171	0/181
Seyyedi (42)	2024	Iran	Cross-sectional	Semi-Nested PCR	FFPE tissue	TAg	3/13	
Kadhum (43)	2024	Iraq	Cross-sectional	qPCR	FFPE tissue	TAg	0/74	
Elyasi (44)	2021	Iran	Cross-sectional	Semi-Nested PCR	FFPE tissue	TAg	33/50	
TIABI (45)	2021	Morocco	Cross-sectional	PCR	Fresh tissue	Not reported	12/50	
ANZIVINO (46)	2015	Italy	Cross-sectional	qPCR	Fresh tissue	TAg	6/26	
Mischitelli (47)	2015	Italy	Cross-sectional	qPCR	Fresh tissue	Not reported	31/71	
Rodríguez (48)	2015	Chile	Cross-sectional	qPCR	FFPE tissue	TAg	6/69	
Akgul (49)	2012	Germany	Cross-sectional	qPCR	FFPE tissue	Not reported	1/85	
Sfanos (50)	2008	USA	Cross-sectional	Nested-PCR	Fresh tissue	TAg	1/200	
Balis (51)	2007	Greece	Cross-sectional	PCR	FFPE tissue and fresh tissue	VP1	8/42	
Das (52)	2004	USA	Cross-sectional	PCR	FFPE tissue	TAg	16/21	
Zambrano (53)	2002	USA	Cross-sectional	Nested-PCR	FFPE tissue and fresh tissue	TAg	2/7	

### 3.2 Quality assessment results

All 23 studies included in this meta-analysis were observational studies (11 case-control studies (19, 27–29, 35–41) and 12 cross-sectional studies (42–53)). The quality assessment results show that 5 studies (NOS score 7–9 or modified NOS score 8–10) being rated as high-quality studies and 18 studies being rated as medium-quality studies (NOS score 4–6 or modified NOS score 5–7) (**Supplementary Tables S3, S4**).

### 3.3 Meta-analysis

A total of 23 studies were included in the meta-analysis, comprising 1,629 prostate cancer tissue samples overall; among these, 11 case-control studies contributed 921 PCa cases and 941 controls for the association analysis.

#### 3.3.1 Prevalence of BKPyV in PCa tissues

**Figure 2:**
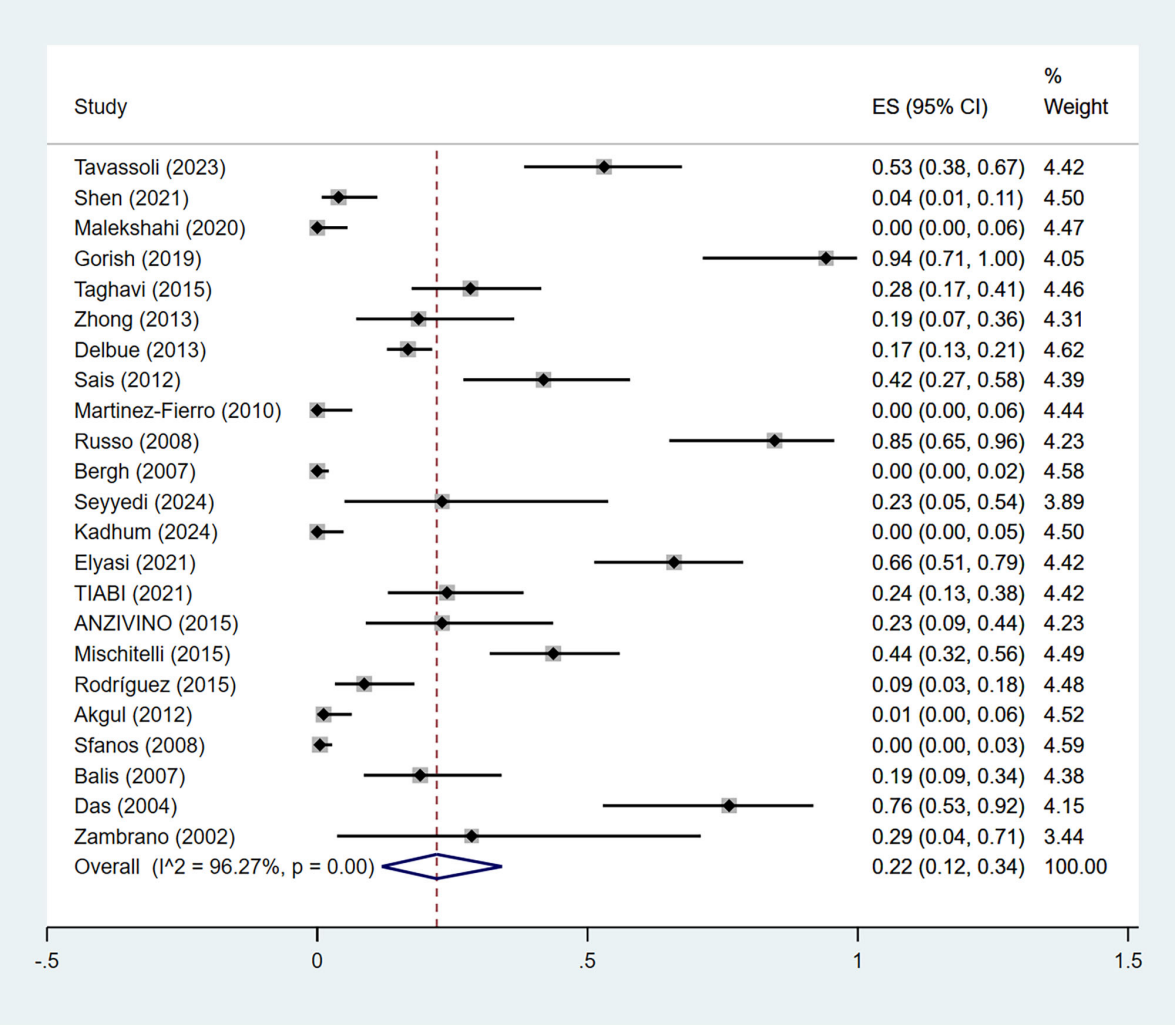
The prevalence of BKPyV in PCa tissue was studied by the random-effect meta-analysis. Forest plot of the pooled prevalence of BKPyV DNA in PCa tissues.

Across 23 studies including 1,629 PCa tissue samples, the overall pooled prevalence of BKPyV in PCa tissues was calculated. The pooled prevalence of BKPyV in PCa tissues was 22% (95% CI: 12–34%). There was a significant heterogeneity between the studies (I^2^ = 96.27%, *p* < 0.01) (**Figure 2**). The 95% PI was 0–84%, indicating that the prevalence in a future comparable study could vary widely. Sensitivity analysis was performed by sequentially omitting individual studies. The results showed that the pooled prevalence remained stable, indicating that the high heterogeneity was not primarily driven by any single study (**Supplementary Figure S1**) (**Supplementary Table S5**).

#### 3.3.2 BKPyV prevalence stratified by detection method

**Figure 3:**
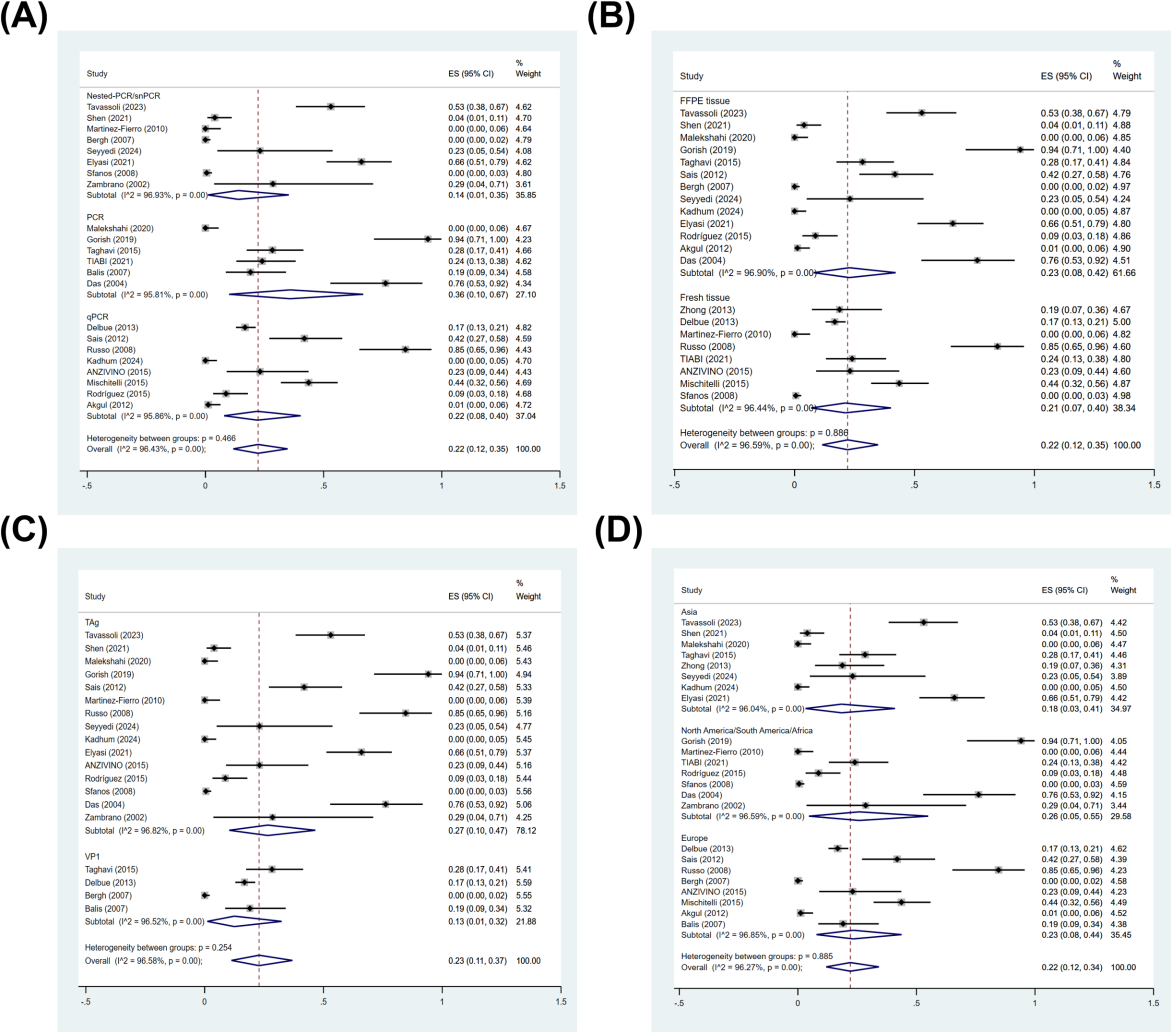
Subgroup analyses of BKPyV prevalence in PCa tissues. Forest plots showing pooled prevalence estimates stratified by: **(A)** detection method (Nested-/Semi-nested PCR, PCR, qPCR), **(B)** specimen type (FFPE tissue, fresh tissue), **(C)** PCR target region (TAg, VP1), and **(D)** study location (Asia, Europe, North America/South America/Africa).

Among studies with classifiable data for detection method, the pooled prevalence analysis included 8 Nested-PCR/Semi-Nested PCR (snPCR) studies (n = 621), 6 PCR studies (n = 254), and 8 qPCR studies (n = 722). In the subgroup analysis by detection method, the pooled prevalence was 14% (95% CI: 1–35%; I^2^ = 96.93%) for Nested-PCR/snPCR, 36% (95% CI: 10–67%; I^2^ = 95.81%) for PCR, and 22% (95% CI: 8–40%; I^2^ = 95.86%) for qPCR (**Figure 3A**). However, Cochran’s Q test showed that there was no significant difference between methods (Qb = 1.53, df = 2, *p* = 0.47) (**Supplementary Table S5**).

#### 3.3.3 BKPyV prevalence stratified by specimen type

Among studies with classifiable data for specimen type, the pooled prevalence analysis included 13 FFPE tissue studies (n = 708), and 8 fresh tissue studies (n = 788). In the subgroup analysis by specimen type, the pooled prevalence was 23% (95% CI: 8–42%; I^2^ = 96.90%) for FFPE tissue and 21% (95% CI: 7–40%; I^2^ = 96.44%) for fresh tissue (**Figure 3B**). Cochran’s Q test, however, found no significant difference between FFPE and fresh tissue specimens (Qb = 0.02, df = 1, *p* = 0.89) (**Supplementary Table S5**).

#### 3.3.4 BKPyV prevalence stratified by molecular target region of the PCR

Among studies with classifiable data for PCR target region, the pooled prevalence analysis included 15 TAg studies (n = 790), and 4 VP1 studies (n = 601). In the subgroup analysis by molecular target region of the PCR, the pooled prevalence was 27% (95% CI: 10–47%; I^2^ = 96.82%) for TAg and 13% (95% CI: 1–32%; I^2^ = 96.52%) for VP1 (**Figure 3C**). While TAg and VP1 regions yielded distinct pooled prevalence estimates, Cochran’s Q test revealed no statistically meaningful distinction between these PCR target regions (Qb = 1.30, df = 1, *p* = 0.25) (**Supplementary Table S5**).

#### 3.3.5 BKPyV prevalence stratified by study location

Among studies with classifiable data for study location, the pooled prevalence analysis included 8 Asia studies (n = 418), 8 Europe studies (n = 792), and 7 North America/South America/Africa studies (n = 419). In the subgroup analysis by study location, the pooled prevalence was 18% (95% CI: 3–41%; I^2^ = 96.04%) for Asia, 26% (95% CI: 5–55%; I^2^ = 96.59%) for North America/South America/Africa, and 28% (95% CI: 8–44%; I^2^ = 96.85%) for Europe (**Figure 3D**). Cochran’s Q test indicated no significant difference between study location (Qb = 0.24, df = 2, *p* = 0.88) (**Supplementary Table S5**).

#### 3.3.6 BKPyV prevalence stratified by Gleason score

**Figure 4:**
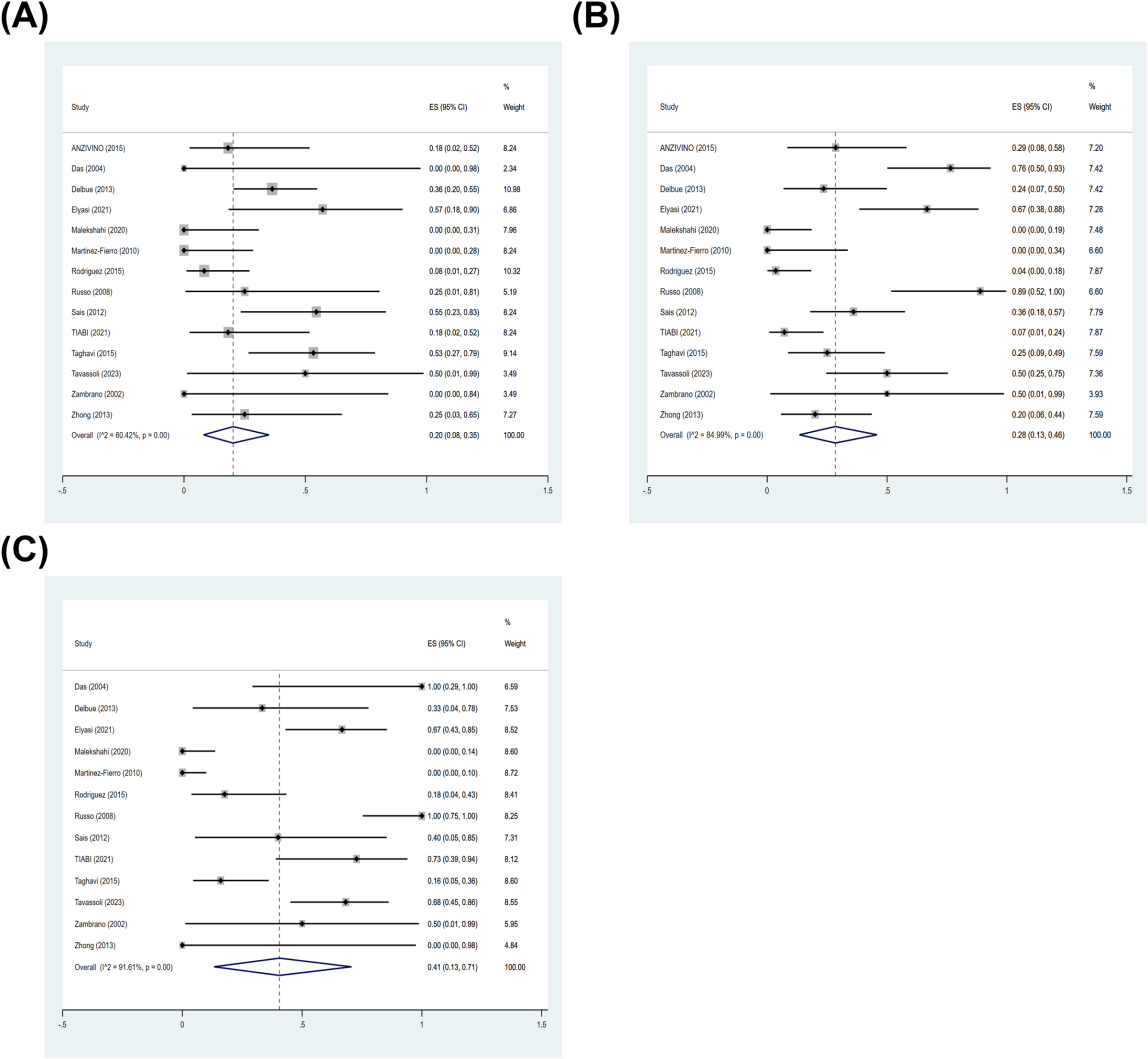
BKPyV prevalence in PCa tissues stratified by Gleason score. Forest plots of pooled prevalence of BKPyV DNA in PCa tissues by Gleason score: **(A)** Gleason score 6, **(B)** Gleason score 7, and **(C)** Gleason score 8–10.

**Figure 5:**
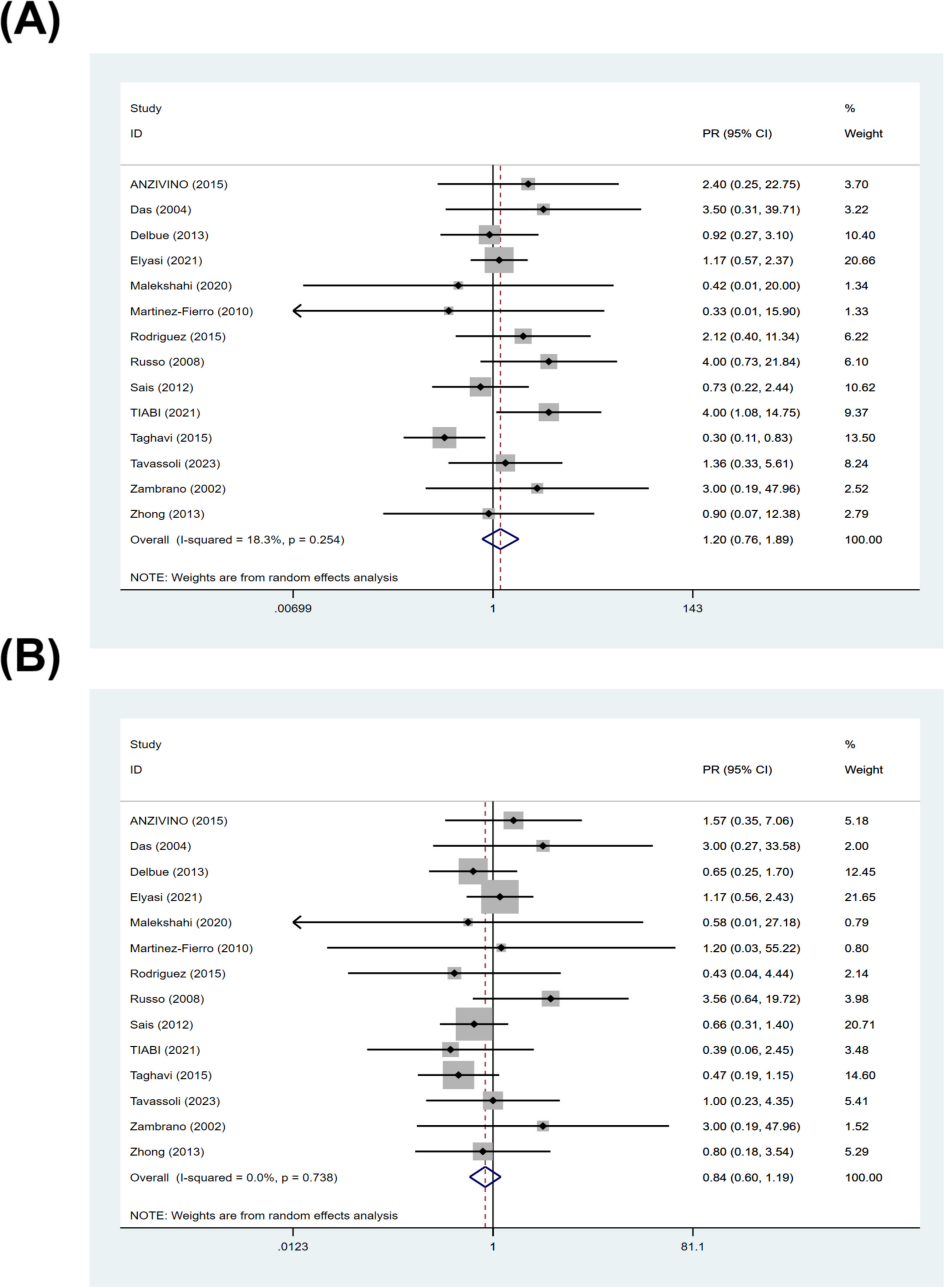
Inter-study contrasts of BKPyV prevalence across Gleason score (PRs). **(A)** PR comparing Gleason score 8–10 vs 6; **(B)** PR comparing Gleason score 7 vs 6.

Among studies with classifiable data for Gleason score, 14 studies contributed data to Gleason score subgroup. In the subgroup analysis stratified by Gleason score, the pooled prevalence of BKPyV in PCa tissues was 20% (95% CI: 8–35%; I²= 60.42%) for Gleason score 6 (**Figure 4A**), 28% (95% CI: 13–46%; I²= 84.99%) for Gleason score 7 (**Figure 4B**), and 41% (95% CI: 13–71%; I²= 91.61%) for Gleason score 8–10 (**Figure 4C**). Although the prevalence was higher in tumors with higher Gleason scores, the overlap of CIs between strata was high and heterogeneity remained substantial across strata. To avoid violating the independence assumption when individual studies contributed multiple Gleason strata, we additionally pooled within-study PRs using Gleason score 6 as the reference. The pooled PR was 1.20 (95% CI: 0.79–1.89; I²= 18.3%) for Gleason score 8–10 vs 6 (**Figure 5A**), and 0.84 (95% CI: 0.60–1.19; I²= 0.0%) for Gleason score 7 vs 6 (**Figure 5B**). These results show that there is no statistically significant difference in the prevalence of BKPyV with different Gleason scores based on the comparison within the study.

#### 3.3.7 Association between BKPyV infection and PCa

**Figure 6:**
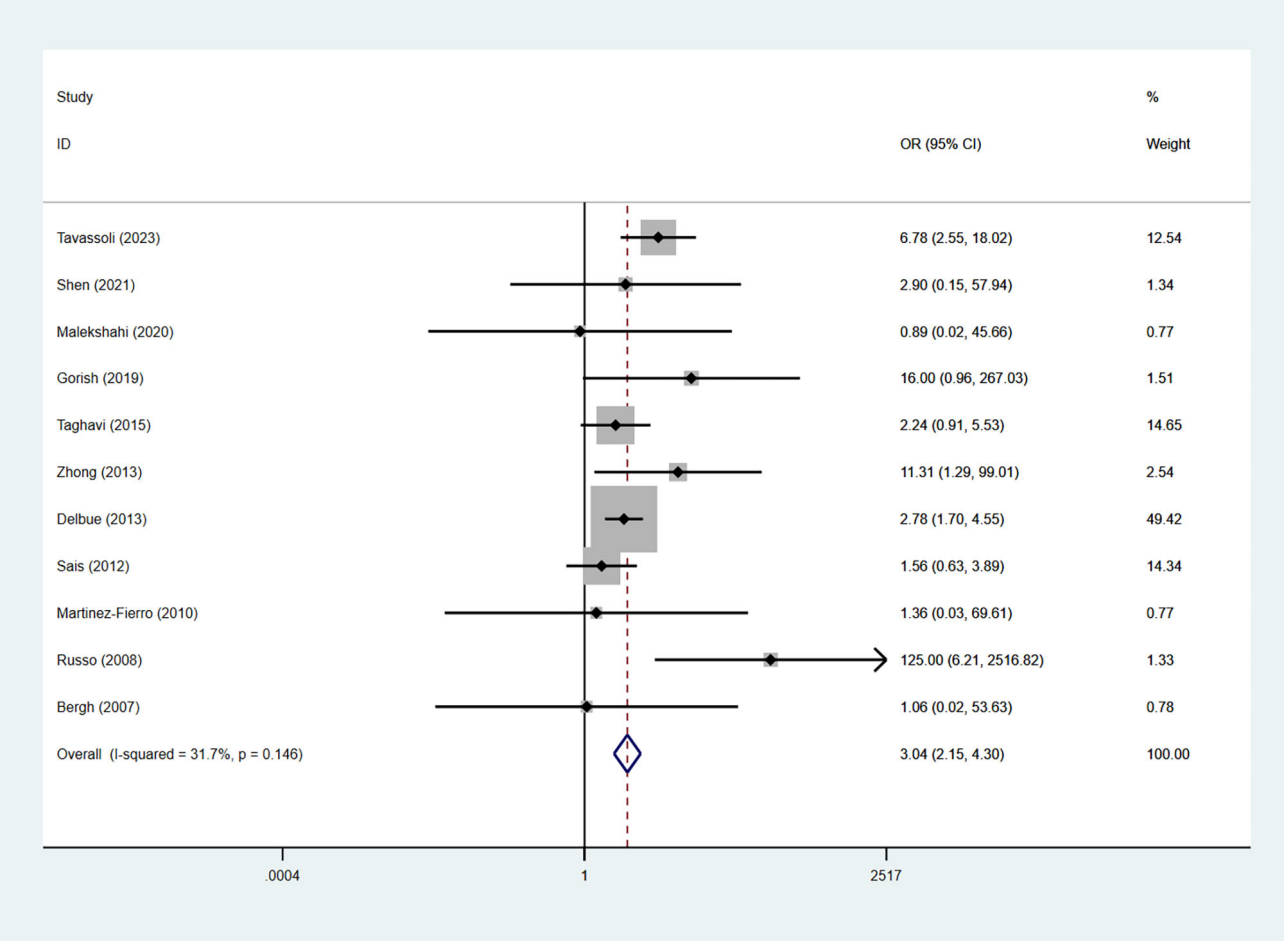
The association between BKPyV infection and PCa was studied by the fixed-effect meta-analysis. Forest plot showing the association between BKPyV infection and PCa.

We used a fixed-effects model to calculate the pooled OR. As shown in the Figure, PCa patients showed a significantly higher rate of BKPyV infection compared to non-cancerous controls. The association analysis included 11 case-control studies comprising 921 PCa cases and 941 controls. Among these studies, 10 studies used BPH patients as controls, and another study included controlled subjects who underwent transrectal biopsy or transurethral prostatectomy but had no histopathological evidence of PCa, these controls were mainly individuals with BPH-related clinical indications (e.g., refractory urinary retention, recurrent urinary infection, macrohematuria, bladder calculi, renal insufficiency, or prostatic diverticuli) (39). Specifically, the presence of BKPyV was associated with a 3.04-fold increase in the odds of PCa (pooled OR = 3.04, 95% CI: 2.15–4.30, 95% PI: 1.02–11.40) (**Figure 6**). There was no evidence of obvious heterogeneity between these studies (I^2^ = 31.7%, *p* = 0.146) (**Supplementary Table S6**). The researchers first found asymmetry through visual examination of the funnel plot, which initially suggested that there might be publication bias (**Supplementary Figure S2**). However, the subsequent Egger’s test failed to show statistical significance (p = 0.370) (**Supplementary Figure S3A**). Nevertheless, given the relatively small number of articles that meet the inclusion criteria, the statistical power of the test may not be sufficient to completely eliminate publication bias. Therefore, we used the Trim and Fill method for further analysis. No missing studies were identified in this analysis, and the pooled estimate remained unchanged (**Supplementary Figure S3B**). This further confirmed the robustness of the results and indicated that the results are not significantly affected by publication bias. Our sensitivity analysis showed that individual reports have no significant impact on the overall pooled effect estimate (**Supplementary Figure S4**).

#### 3.3.8 Association between BKPyV infection and PCa based on detection method

**Figure 7:**
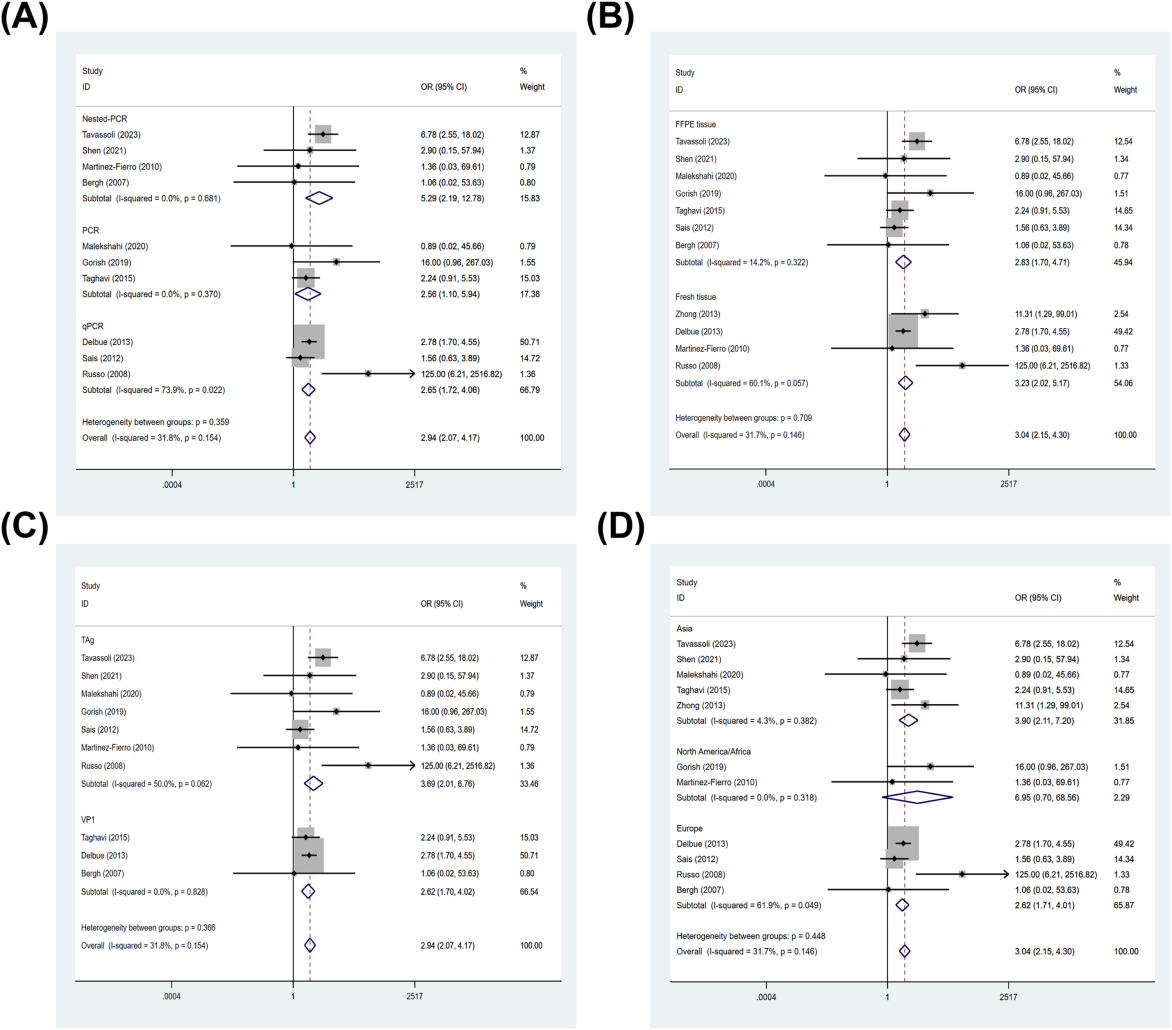
Subgroup analyses of the association between BKPyV infection and PCa. Forest plots of pooled ORs stratified by: **(A)** detection method (Nested-/Semi-nested PCR, PCR, qPCR), **(B)** specimen type (FFPE tissue, fresh tissue), **(C)** PCR target region (TAg, VP1), and **(D)** study location (Asia, Europe, North America/Africa).

Among case-control studies with classifiable data for detection method, 4 Nested-PCR studies contributed 351 PCa cases and 335 controls, 3 PCR studies contributed 141 PCa cases and 121 controls, and 3 qPCR studies contributed 397 PCa cases and 435 controls. In the subgroup analysis stratified by detection method, BKPyV infection was significantly associated with PCa across all three assay categories. The pooled OR was 5.29 (95% CI: 2.19–12.78; I²= 0.0%) for Nested-PCR, 2.56 (95% CI: 1.10–5.94; I²= 0.0%) for PCR, and 2.65 (95% CI: 1.72–4.06; I²= 73.9%) for qPCR (**Figure 7A**). Cochran’s Q test showed that the subgroup differences between each detection method are not statistically significant (Qb = 2.05, df = 2, *p* = 0.359) (**Supplementary Table S6**).

#### 3.3.9 Association between BKPyV infection and PCa based on specimen type

mong case-control studies with classifiable data for specimen type, 7 FFPE tissue studies contributed 480 PCa cases and 419 controls, and 4 fresh tissue studies contributed 441 PCa cases and 522 controls. Subgroup analysis by specimen type showed a significant association between BKPyV infection and PCa in both FFPE tissues and fresh tissues. The pooled OR was 2.83 (95% CI: 1.70–4.71; I²= 14.2%) for FFPE tissues, and 3.23 (95% CI: 2.02–5.17; I²= 60.1%) for fresh tissues (**Figure 7B**). The heterogeneity between subgroups was not significant (Qb = 0.14, df = 1, *p* = 0.709) (**Supplementary Table S6**).

#### 3.3.10 Association between BKPyV infection and PCa based on molecular target region of the PCR

Among case-control studies with classifiable data for molecular target region of the PCR, 7 TAg based studies contributed 330 PCa cases and 265 controls, and 3 VP1 based studies contributed 559 PCa cases and 626 controls. When stratified by PCR target region, BKPyV remained significantly associated with PCa regardless of whether assays targeted TAg or VP1. The pooled OR was 3.69 (95% CI: 2.01–6.76; I²= 50.0%) for TAg-based assays, and 2.62 (95% CI: 1.70–4.02; I²= 0.0%) for VP1-based assays (**Figure 7C**). No significant difference was observed between target-region subgroups (Qb = 0.82, df = 1, *p* = 0.366) (**Supplementary Table S6**).

#### 3.3.11 Association between BKPyV infection and PCa based on study location

Among case-control studies with classifiable data for study location, 5 studies from Asia contributed 281 PCa cases and 246 controls, 4 from Europe contributed 568 PCa cases and 616 controls, and 2 from North America/Africa contributed 72 PCa cases and 79 controls. In subgroup analyses by study location, BKPyV infection was associated with PCa across regions, although precision differed by subgroup size. The pooled OR was 3.90 (95% CI: 2.11–7.20; I²= 4.3%) for Asia, 6.95 (95% CI: 0.70–68.56; I²= 0.0%) for North America/Africa, and 2.62 (95% CI: 1.71–4.01; I²= 61.9%) for Europe (**Figure 7D**). Cochran’s Q test suggested no statistically significant differences between geographic subgroups (Qb = 1.60, df = 2, p = 0.448) (**Supplementary Table S6**).

#### 3.3.12 Association between BKPyV infection and PCa based on Gleason score

**Figure 8:**
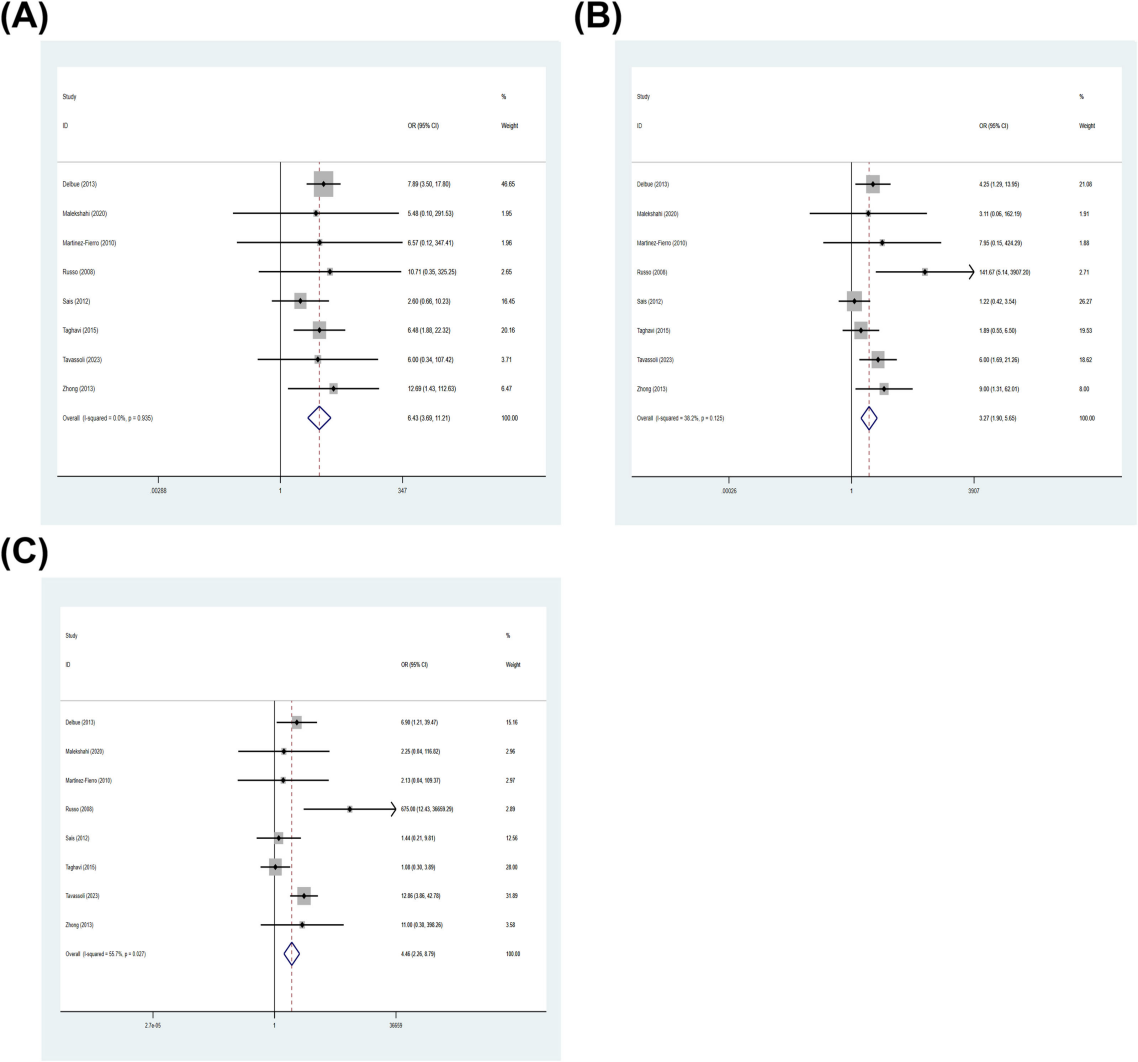
Association between BKPyV positivity and PCa stratified by Gleason score. Forest plots of pooled ORs for the association between BKPyV infection and PCa within Gleason score: **(A)** Gleason score 6, **(B)** Gleason score 7, and **(C)** Gleason score 8–10.

Among studies with classifiable Gleason score data, 8 case-control studies contributed data to Gleason score subgroup. In the subgroup analysis stratified by Gleason score, BKPyV infection was significantly associated with PCa across all grade categories. The pooled OR was 6.43 (95% CI: 3.69–11.21) for Gleason score 6, with no evidence of heterogeneity (I²= 0.0%, *p* = 0.935) (**Figure 8A**). For Gleason score 7, the pooled OR was 3.27 (95% CI: 1.90–5.65), with moderate heterogeneity (I²= 38.2%, *p* = 0.125) (**Figure 8B**). For Gleason score 8–10, the pooled OR was 4.46 (95% CI: 2.26–8.79), with moderate heterogeneity (I²= 55.7%, *p* = 0.027) (**Figure 8C**) (**Supplementary Table S6**).

**Figure 9:**
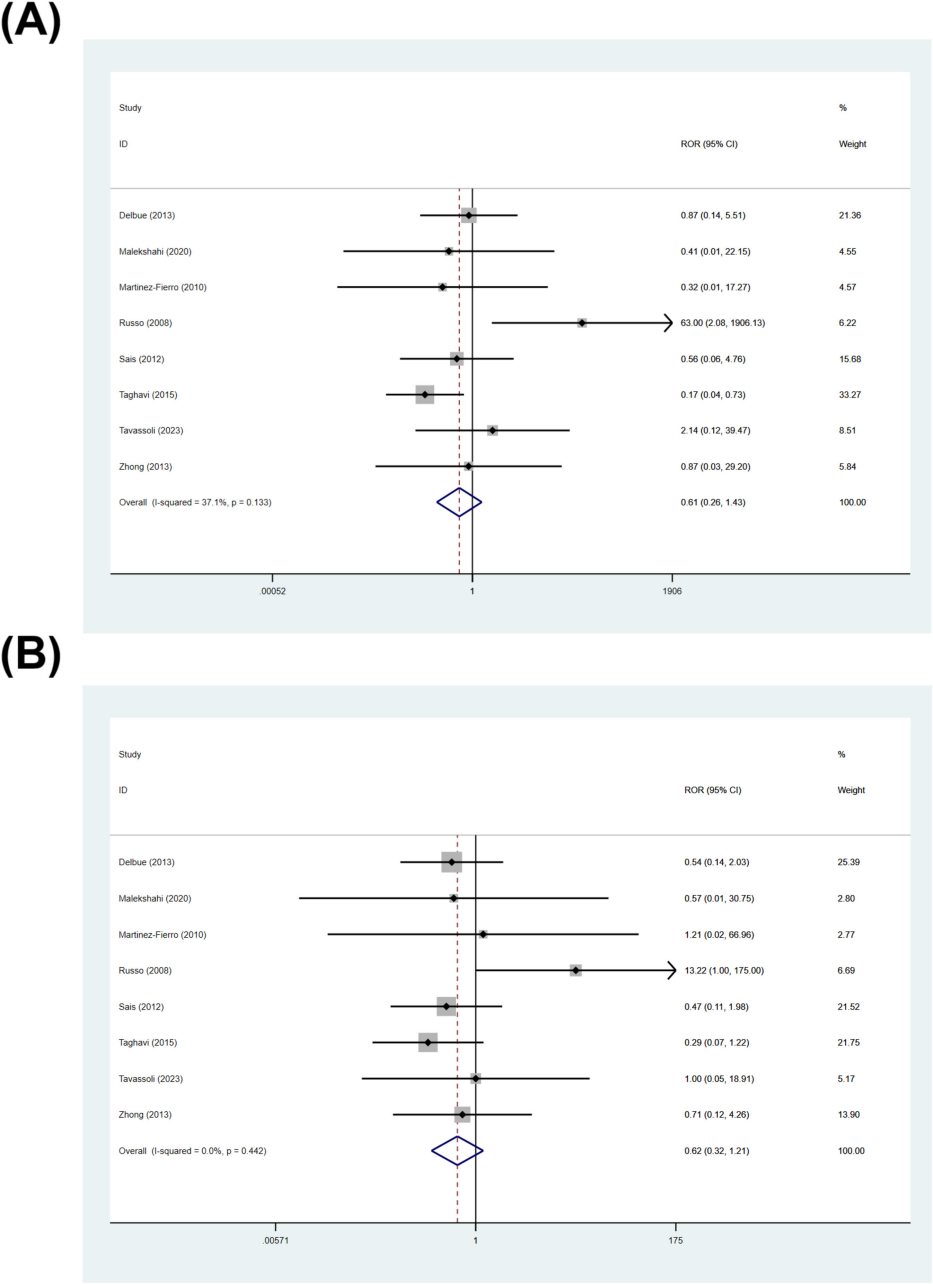
Inter-study contrasts of association strength across Gleason score (RORs). **(A)** ROR comparing Gleason score 8–10 vs 6; **(B)** ROR comparing Gleason score 7 vs 6.

Because some primary studies reported multiple Gleason score strata while sharing the same control group, treating each stratum as an independent comparison may violate the independence assumption. Therefore, we conducted an inter-study comparison by pooled ROR to explore whether the Gleason score affects the association between BKPyV and prostate cancer. The pooled ROR was 0.61 (95% CI: 0.26–1.43; I²= 37.1%, *p* = 0.133) for Gleason score 8–10 vs 6 (**Figure 9A**), and 0.62 (95% CI: 0.32–1.21; I²= 0.0%, *p* = 0.442) for Gleason score 7 vs 6 (**Figure 9B**). Based on the within-study comparative analysis, there was no statistically significant difference in the association between BKPyV and PCa between different Gleason score.

## 4 Discussion

This systematic review and meta-analysis synthesizes the current research on the association between BKPyV infection and PCa. This study has two major findings. First, BKPyV DNA could be detected in many PCa tissues (pooled prevalence 22%), but the estimated values vary significantly between studies, and the PI is extremely wide (0% – 84%), indicating that future studies under similar conditions may still observe significantly different prevalence rates. More importantly, case-control analyses reveal a consistent and significant risk association, demonstrating that BKPyV positivity in prostate tissue is associated with a roughly threefold increased odds of PCa (pooled OR 3.04), with only low-to-moderate heterogeneity. Although the heterogeneity of the association analysis was modest, subgroup analyses were pre-set to test robustness and evaluate the impact of grouping factors on the results. These findings support a robust association and there is a reason to conduct further research on the possibility of BKPyV as a potential synergistic factor for PCa. It is worth noting that in the research using Nested-PCR, the pooled OR was numerically higher than the research using conventional PCR or qPCR, although the difference between the methods has not reached a statistically significant level. One possible explanation is that Nested-PCR may have higher sensitivity to detect low-copy or focal distribution of viral DNA in prostate tissue. At the same time, the interpretation of this phenomenon should be cautious, because our analysis is based on the specific detection method for BKPyV rather than broad polyomavirus profiling. Nevertheless, the finding raises the possibility that the polyomavirus milieu in prostate tissue may be more complex than detection of BKPyV alone suggests, and that coexisting or related human polyomaviruses merit further investigation in future studies using highly specific multiplex or sequencing-based approaches.

The significant heterogeneity in prevalence is likely multifactorial and reflects both biological and methodological variability. BKPyV infection in the urogenital tract is often latent or low-level, which can translate into focal and patchy intraprostatic detection (52). Thus, the detection rate may be highly sensitive to sampling depth, tissue processing, amplicon length, primer/probe design, and laboratory contamination control, particularly when viral burden is low and sequence polymorphisms affect assay matching (54). Consistent with this interpretation, the subgroup analysis by specimen type, detection method and PCR target region failed to fully explain the variability between studies, indicating that other unmeasured technical factors and biological differences together led to the observed dispersion. It is worth noting that our subgroup analysis based on study location did not detect statistically significant differences in prevalence between regions, which shows that the vast geographical scope alone is unlikely to be the main source of heterogeneity. However, BKPyV exhibits finer-scale geographic genotype structure. Zhong et al. reported that while subtype I is ubiquitous worldwide, subtype IV shows a skewed distribution with higher prevalence in East Asian populations (55), we speculate that regional differences in the distribution of BKPyV subtypes may be an unmeasured contributor to the observed heterogeneity.

Regarding tumor aggressiveness, our analyses did not provide strong evidence that BKPyV detection or the strength of association differs by Gleason score. Although the point estimates of prevalence and ORs are often higher in the Gleason score 8–10 category, the inter-study comparison (PR and ROR) that maintain research independence does not show a statistically significant difference between the Gleason categories. In general, the existing evidence does not support the statistically detectable effect of the Gleason score; however, given the limited number of reports and the small sample size, the possibility of a slight difference cannot be ruled out. These findings also highlight the need to report clinical pathology metadata (such as PSA levels, tumor staging and treatment background) more uniformly in original studies.

A key biological question raised by our meta-analysis is what PCR detectable BKPyV DNA represents biologically in prostate tissue and why does its detectability show such a huge difference between different cohorts. The persistent presence of BKPyV in the urogenital tract is typically latent and low level (14, 16), which may lead to its focal, patchy distribution and highly dependent on the local tissue environment. Although there are significant differences in BKPyV prevalence between different cohorts, the BKPyV positive rate is more stable in prostate cancer patients than that of the non-cancer control group. PCa is often described as an immunological cold malignant tumor, and its tumor microenvironment has immunosuppressive characteristics (56, 57). In some lesions, the weakening of virus specific immune surveillance function may lead to persistent presence or intermittent activity at low levels of the virus. Research on the correlation of supporting viral targeted immunity in the prostate shows that there is a significant difference in the specific immune response of TAg between patients with BKPyV positive PCa and patients with BPH (39). Moreover, inflammatory and stress signals within the tumor microenvironment may influence BKPyV transcriptional control: TGF-β can regulate BKPyV early gene transcription (58), TNF-α/NF-κB signaling has been shown to enhance BKPyV promoter activity and replication in cellular models (59), and hypoxia-related pathways have been linked to HIF-1α–mediated stimulation of BKPyV replication (60). Although these observations are not prostate specific, they provide biologically plausible explanations for how heterogeneous microenvironmental states could contribute to the marked dispersion of PCR detectability across studies. This interpretation is also compatible with our finding that BKPyV detection did not show a clear gradient across Gleason score categories, which may suggest that detectability can reflect tumor associated tissue context without necessarily tracking with cancer severity.

Another mechanism to consider is the genome integration of BKPyV DNA in the host genome. In human tissues, BKPyV is generally considered to persist mainly in the form of latent episomal virus rather than as a constitutively integrated form (13, 14). However, the phenomenon of BKPyV integration into host chromosome DNA has been documented in BKPyV-associated urothelial carcinomas, particularly in renal transplant recipients (61, 62). In these tumors, integration is often accompanied by the destruction of late viral genes and persistent TAg expression, which is consistent with the phenomenon of loss of productive replication but retention of transforming potential (61). More recent genome wide analyses have further characterized integration sites and suggested nucleotide level mechanisms underlying BKPyV integration in these tumors (63, 64). This mechanism is relevant to the interpretation of our findings because all studies included in the present meta analysis relied on PCR-based detection of BKPyV DNA. Although PCR is sensitive to viral sequence detection, it cannot determine whether the detected viral DNA is episomal, integrated, defective, or transcriptionally active. Similarly, if the result of the late region target such as VP1 is negative, if the region has been fractured in the integration event, the possibility of previous integration cannot be ruled out (61, 63). Therefore, genomic integration is a biologically reasonable mechanism that may help explain why some tumors retain BKPyV sequences or evidence of transforming early region signals without clear evidence of productive infection. At the same time, direct evidence of BKPyV integration in prostate cancer remains limited, so this possibility should be interpreted cautiously.

Overall, our results are best explained by considering at least three non-mutually exclusive hypotheses. First of all, the hit-and-run hypothesis is still biologically rational because BKPyV TAg can disrupt the p53 and pRb pathways, thereby promoting cell cycle dysregulation and genomic instability during early stages of transformation (20, 21, 25). Under this hypothesis, limited or transient viral activity could contribute to tumor initiation, after which viral genomes may become partially lost, degraded, or fall below detection thresholds (21, 25, 42). However, because BKPyV DNA can still be detected in some PCa tissues in our meta-analysis, it is difficult to fully explain all positive tumor cases by the hit-and-run hypothesis alone. Second, BKPyV detectability may in some cases primarily reflect a microenvironment driven phenomenon, in which local immune suppression and tumor-associated signaling facilitate viral persistence or intermittent replication in prostate tumors without requiring a direct causal role in carcinogenesis. Under this hypothesis, BKPyV positivity would be better interpreted as an accompanying pathological marker rather than a specific driver of the pathophysiology of PCa. This interpretation is compatible with the significant heterogeneity in BKPyV prevalence across studies, the overall positive association with PCa risk, and we did not observe a clear gradient change between the Gleason score categories in the study comparison. In other words, BKPyV may be preferentially detectable in tumor-associated tissue environments without necessarily tracking with cancer severity. Third, a mixed phenomenon may also be considered, in which BKPyV and the tumor microenvironment mutually sustain each other over time through a long and complex process involving alternating phases of low-level viral replication, partial immune control, and renewed detectability. Such a hypothesis could better accommodate the combination of a relatively stable positive association with PCa risk and substantial variability in viral detection across cohorts observed in our meta-analysis. Based on the existing data, we can determine neither the temporal sequence nor the causal relationship, and we cannot clearly distinguish among these hypotheses. Therefore, BKPyV should be regarded as a possible cofactor in prostate carcinogenesis or as a context-dependent marker shaped by the tumor microenvironment.

Future studies should give priority to standardized molecular detection protocols, including harmonized primer and probe sets, PCR procedures, and related laboratory workflows. Detailed clinicopathological variables, such as Gleason score, tumor stage, PSA level, immune infiltration, and treatment history, should also be reported more systematically. Longitudinal studies or well annotated biobanks would be particularly valuable. These approaches may help clarify whether BKPyV is detected before tumor development, intermittently during tumor evolution, or only enriched in specific immune or molecular subtypes of prostate cancer, thereby revealing whether BKPyV acts as a pathogenic cofactor, a marker of immune dysregulation, or both.

## 5 Limitations

This study has several limitations. First, despite subgroup analyses by specimen type, detection method, PCR target region, study location, and Gleason score, substantial residual heterogeneity remained, particularly in the prevalence meta-analysis. This heterogeneity likely reflects unmeasured methodological and biological differences, including tissue processing, DNA quality, assay design, and contamination control. Second, several subgroup analyses were based on a limited number of studies, and most primary reports lacked detailed information on age, PSA level, tumor stage, treatment history, immune context, and other potential confounders, which restricted more refined stratified analyses. Third, all included studies were observational, so the pooled estimates support association rather than causation and do not establish the temporal sequence between BKPyV detection and prostate carcinogenesis. Fourth, in order to ensure the comparability of methodology, the meta-analysis is limited to PCR-based research. Although PCR is sensitive for detecting viral sequences, it cannot determine whether the detected viral DNA is episomal, integrated, defective, or transcriptionally active; nor can it define viral localization or protein expression, so potentially informative ISH/IHC-based evidence was not incorporated into the pooled analysis. Finally, some Gleason score subgroup analyses involved correlated data structures because multiple tumor strata shared the same control group; although within-study contrasts were used to reduce this bias, these results should still be interpreted cautiously.

## 6 Conclusion

Our findings indicate that BKPyV positivity is more common in PCa tissues than in control tissues and is positively associated with PCa. Although the heterogeneity of the prevalence estimates limits its universality, the positive association was generally stable in the subgroup analysis according to detection methods, sample types, molecular target region, study location and Gleason scores. These findings suggest a possible role for BKPyV in PCa, but do not establish causality. The observed results are related to three possible hypotheses, including hit-and-run, microenvironment driven phenomenon, and mixed phenomenon. Future studies integrating standardized molecular detection protocols, viral integration analyses, and detailed clinicopathological annotation will be essential to clarify causality and to determine whether BKPyV has value as a risk-related biomarker in PCa.

## Data Availability

The original contributions presented in the study are included in the article/**Supplementary Material**. Further inquiries can be directed to the corresponding author.

## Supplementary material

Supplementary Figure S1 Leave-one-out sensitivity analysis for pooled prevalence. Influence analysis showing the pooled prevalence estimate after sequential omission of each individual study.

Supplementary Figure S2 Funnel plot for the meta-analysis of the association between BKPyV infection and PCa.

Supplementary Figure S3 Evaluation of publication bias for the association between BK polyomavirus infection and prostate cancer. **(A)** Egger’s test plot; **(B)** Results of the Trim and Fill analysis.

Supplementary Figure S4 Leave-one-out sensitivity analysis for pooled OR. Influence analysis showing the pooled OR estimate after sequential omission of each individual study.

## References

[1] Wang L Lu B He M Wang Y Wang Z Du L . Prostate cancer incidence and mortality: Global status and temporal trends in 89 countries from 2000 to 2019. Front Public Health. (2022) 10:811044. doi: 10.3389/fpubh.2022.811044. PMID: 35252092 PMC8888523

[2] Filho A Laversanne M Ferlay J Colombet M Piñeros M Znaor A . The GLOBOCAN 2022 cancer estimates: Data sources, methods, and a snapshot of the cancer burden worldwide. Int J Cancer. (2025) 156:1336–46. doi: 10.1002/ijc.35278. PMID: 39688499

[3] Cornford P van den Bergh R Briers E Van den Broeck T Brunckhorst O Darraugh J . EAU-EANM-ESTRO-ESUR-ISUP-SIOG guidelines on prostate cancer-2024 update. Part I: Screening, diagnosis, and local treatment with curative intent. Eur Urol. (2024) 86:148–63. doi: 10.1016/j.eururo.2024.03.027. PMID: 38614820

[4] Raychaudhuri R Lin D Montgomery R . Prostate cancer: A review. JAMA. (2025) 333:1433–46. doi: 10.1001/jama.2025.0228. PMID: 40063046

[5] James N Tannock I N’Dow J Feng F Gillessen S Ali S . The Lancet Commission on prostate cancer: Planning for the surge in cases. Lancet. (2024) 403:1683–722. doi: 10.1016/S0140-6736(24)00651-2. PMID: 38583453 PMC7617369

[6] Center M Jemal A Lortet-Tieulent J Ward E Ferlay J Brawley O . International variation in prostate cancer incidence and mortality rates. Eur Urol. (2012) 61:1079–92. doi: 10.1016/j.eururo.2012.02.054. PMID: 22424666

[7] Allott E Masko E Freedland S . Obesity and prostate cancer: Weighing the evidence. Eur Urol. (2013) 63:800–9. doi: 10.1016/j.eururo.2012.11.013. PMID: 23219374 PMC3597763

[8] Peisch S Van Blarigan E Chan J Stampfer M Kenfield S . Prostate cancer progression and mortality: A review of diet and lifestyle factors. World J Urol. (2017) 35:867–74. doi: 10.1007/s00345-016-1914-3. PMID: 27518576 PMC5472048

[9] Reiter-Brennan C Dzaye O Al-Mallah M Dardari Z Brawner C Lamerato L . Fitness and prostate cancer screening, incidence, and mortality: Results from the Henry Ford Exercise Testing (FIT) Project. Cancer. (2021) 127:1864–70. doi: 10.1002/cncr.33426. PMID: 33561293

[10] Guzha B Matubu A Nyandoro G Mubata H Moyo E Murewanhema G . The impact of DNA tumor viruses in low-to-middle income countries (LMICS): A literature review. Tumour Virus Res. (2024) 18:200289. doi: 10.1016/j.tvr.2024.200289. PMID: 38977263 PMC11298656

[11] Moore P Chang Y . Why do viruses cause cancer? Highlights of the first century of human tumour virology. Nat Rev Cancer. (2010) 10:878–89. doi: 10.1038/nrc2961. PMID: 21102637 PMC3718018

[12] Kiś J Sikora D Jarosz M Polz-Dacewicz M . JC polyomavirus in prostate cancer-friend or foe? Cancers. (2025) 17:1725. doi: 10.3390/cancers17101725. PMID: 40427223 PMC12109926

[13] Ambalathingal G Francis R Smyth M Smith C Khanna R . BK polyomavirus: Clinical aspects, immune regulation, and emerging therapies. Clin Microbiol Rev. (2017) 30:503–28. doi: 10.1128/CMR.00074-16. PMID: 28298471 PMC5355639

[14] Zhou X Zhu C Li H . BK polyomavirus: Latency, reactivation, diseases and tumorigenesis. Front Cell Infect Microbiol. (2023) 13:1263983. doi: 10.3389/fcimb.2023.1263983. PMID: 37771695 PMC10525381

[15] Furmaga J Kowalczyk M Zapolski T Furmaga O Krakowski L Rudzki G . BK polyomavirus-biology, genomic variation and diagnosis. Viruses. (2021) 13:1502. doi: 10.3390/v13081502. PMID: 34452367 PMC8402805

[16] Hirsch H Steiger J . Polyomavirus BK. Lancet Infect Dis. (2003) 3:611–23. doi: 10.1016/s1473-3099(03)00770-9. PMID: 14522260

[17] Boukoum H Nahdi I Sahtout W Skiri H Segondy M Aouni M . BK and JC virus infections in healthy patients compared to kidney transplant recipients in Tunisia. Microb Pathog. (2016) 97:204–8. doi: 10.1016/j.micpath.2016.06.015. PMID: 27317859

[18] Ramos E Drachenberg C Wali R Hirsch H . The decade of polyomavirus BK-associated nephropathy: State of affairs. Transplantation. (2009) 87:621–30. doi: 10.1097/TP.0b013e318197c17d. PMID: 19295303

[19] Shen C Tung C Chao C Jou Y Huang S Meng M . The differential presence of human polyomaviruses, JCPyV and BKPyV, in prostate cancer and benign prostate hypertrophy tissues. BMC Cancer. (2021) 21:1141. doi: 10.1186/s12885-021-08862-w. PMID: 34689739 PMC8543972

[20] Moens U Macdonald A . Effect of the large and small T-antigens of human polyomaviruses on signaling pathways. Int J Mol Sci. (2019) 20:3914. doi: 10.3390/ijms20163914. PMID: 31408949 PMC6720190

[21] Levican J Acevedo M León O Gaggero A Aguayo F . Role of BK human polyomavirus in cancer. Infect Agent Cancer. (2018) 13:12. doi: 10.1186/s13027-018-0182-9. PMID: 29632550 PMC5887205

[22] Justice J Needham J Thompson S . BK polyomavirus activates the DNA damage response to prolong S phase. J Virol. (2019) 93:e00130-19. doi: 10.1128/JVI.00130-19. PMID: 31043526 PMC6600189

[23] Baker S Mason A Slip R Skinner K Macdonald A Masood O . Induction of APOBEC3-mediated genomic damage in urothelium implicates BK polyomavirus (BKPyV) as a hit-and-run driver for bladder cancer. Oncogene. (2022) 41:2139–51. doi: 10.1038/s41388-022-02235-8. PMID: 35194151 PMC8862006

[24] Needham J Perritt S Thompson S . Single-cell analysis reveals host S phase drives large T antigen expression during BK polyomavirus infection. PloS Pathog. (2024) 20:e1012663. doi: 10.1371/journal.ppat.1012663. PMID: 39636788 PMC11620372

[25] Tognon M Provenzano M . New insights on the association between the prostate cancer and the small DNA tumour virus, BK polyomavirus. J Transl Med. (2015) 13:387. doi: 10.1186/s12967-015-0754-z. PMID: 26699530 PMC4690311

[26] Theile M Grabowski G . Mutagenic activity of BKV and JCV in human and other mammalian cells. Arch Virol. (1990) 113:221–33. doi: 10.1007/BF01316675. PMID: 2171458

[27] Tavassoli N Vojdani A Salimi-Namin S Khadem-Rezaiyan M Kalantari M Youssefi M . Human BKV large T genome detection in prostate cancer and benign prostatic hyperplasia tissue samples by nested PCR: A case-control study. Mol Biol Res Commun. (2023) 12:149–54. doi: 10.22099/mbrc.2023.47537.1836. PMID: 37886738 PMC10599593

[28] Malekshahi S Yavarian J Salehi R Babaei F Ahmadi S Ghavami N . Epstein-Barr and Bk virus in cancerous and noncancerous prostate tissue. Future Virol. (2020) 15:13–7. doi: 10.2217/fvl-2019-0140

[29] Bergh J Marklund I Gustavsson C Wiklund F Grönberg H Allard A . No link between viral findings in the prostate and subsequent cancer development. Br J Cancer. (2007) 96:137–9. doi: 10.1038/sj.bjc.6603480. PMID: 17117176 PMC2360195

[30] Moher D Liberati A Tetzlaff J Altman D . Preferred reporting items for systematic reviews and meta-analyses: The PRISMA statement. PloS Med. (2009) 6:e1000097. doi: 10.1371/journal.pmed.1000097. PMID: 19621072 PMC2707599

[31] IARC Working Group on the Evaluation of Carcinogenic Risks to Humans . BK POLYOMAVIRUS. In: Malaria and some polyomaviruses (SV40, BK, JC, and merkel cell viruses) Lyon, France: International Agency for Research on Cancer (2013). doi: 10.1097/tp.0000000000004976

[32] Herzog R Álvarez-Pasquin M Díaz C Del Barrio J Estrada J Gil Á . Are healthcare workers’ intentions to vaccinate related to their knowledge, beliefs and attitudes? A systematic review. BMC Public Health. (2013) 13:154. doi: 10.1186/1471-2458-13-154. PMID: 23421987 PMC3602084

[33] Hafizu M Junaid O Sagara R Ravi R Yik F Vythilingam I . Zoonotic brugian filariasis past and present trends in Malaysia: A systematic review and proportionate meta-analysis. Sci Rep. (2025) 15:37323. doi: 10.1038/s41598-025-21328-4. PMID: 41136630 PMC12552516

[34] Barker T Migliavaca C Stein C Colpani V Falavigna M Aromataris E . Conducting proportional meta-analysis in different types of systematic reviews: A guide for synthesisers of evidence. BMC Med Res Methodol. (2021) 21:189. doi: 10.1186/s12874-021-01381-z. PMID: 34544368 PMC8451728

[35] Gorish B Ournasseir M Shammat I . A correlation study of BK polyoma virus infection and prostate cancer among Sudanese patients - immunofluorescence and molecular based case-control study. Infect Agent Cancer. (2019) 14:25. doi: 10.1186/s13027-019-0244-7. PMID: 31548852 PMC6751814

[36] Taghavi A Mohammadi-Torbati P Kashi A Rezaee H Vaezjalali M . Polyomavirus hominis 1(BK virus) infection in prostatic tissues: Cancer versus hyperplasia. Urol J. (2015) 12:2240–4. 10.22037/uj.v12i4.2875 26341765

[37] Zhong S Suzuki M Peng X Shen Z-J Wang X-J Xu T-Y . BK polyomavirus from patients with tissue-derived prostate adenocarcinoma. Future Virol. (2013) 8:313–20. doi: 10.2217/fvl.13.8

[38] Delbue S Matei D Carloni C Pecchenini V Carluccio S Villani S . Evidence supporting the association of polyomavirus BK genome with prostate cancer. Med Microbiol Immunol. (2013) 202:425–30. doi: 10.1007/s00430-013-0304-3. PMID: 23821367

[39] Sais G Wyler S Hudolin T Banzola I Mengus C Bubendorf L . Differential patterns of large tumor antigen-specific immune responsiveness in patients with BK polyomavirus-positive prostate cancer or benign prostatic hyperplasia. J Virol. (2012) 86:8461–71. doi: 10.1128/JVI.00005-12. PMID: 22647697 PMC3421724

[40] Martinez-Fierro M Leach R Gomez-Guerra L Garza-Guajardo R Johnson-Pais T Beuten J . Identification of viral infections in the prostate and evaluation of their association with cancer. BMC Cancer. (2010) 10:326. doi: 10.1186/1471-2407-10-326. PMID: 20576103 PMC2912861

[41] Russo G Anzivino E Fioriti D Mischitelli M Bellizzi A Giordano A . p53 gene mutational rate, Gleason score, and BK virus infection in prostate adenocarcinoma: Is there a correlation? J Med Virol. (2008) 80:2100–7. doi: 10.1002/jmv.21312. PMID: 19040285

[42] Seyyedi N Farhadi A Khajeh F Rafiei Dehbidi G Tandel P Najafi M . Polyomavirus infection in urological cancers: Role of SV40, BKPyV, and JCPyV in prostate, bladder, and renal carcinomas. Future Virol. (2024) 19:573–80. doi: 10.1080/17460794.2025.2457300. PMID: 37339054

[43] Kadhum Abu Gulel H Mezher M . The prevalence of JC and BK viruses among prostate cancer patients in Al-Najaf Al-Ashraf province. Bio Web Conf. (2024) 108:04016. doi: 10.1051/bioconf/202410804016. PMID: 24184215

[44] Elyasi M Makvandi M Ranjbari N Latifi S Kaydani G Shamloo M . Prevalence of human polyomavirus BK virus in prostate cancer patients and benign prostatic hyperplasia: A cross-sectional study on prostate patients referred to Imam Khomeini Hospital in Ahvaz between 2015 and 2017. Jundishapur J Micro. (2021) 14:e115388. doi: 10.5812/jjm.115388

[45] Tiabi I Abumsimir B Saif I Ennaji Y Alaoui Sosse S Laraqui A . Molecular evaluation of BK and MC human polyomavirus as promising prostate cancer markers in Moroccan population. Teikyo Med J. (2021) 44:1749–59.

[46] Anzivino E Rodio D Mischitelli M Bellizzi A Sciarra A Salciccia S . High frequency of JCV DNA detection in prostate cancer tissues. Cancer Genomics Proteomics. (2015) 12:189–200. 26136219

[47] Mischitelli M Bellizzi A Anzivino E Rodio D Sciarra A Gentile V . Results, questions, perspectives of a study on human polyomavirus BK and molecular actors in prostate cancer development. Cancer Genomics Proteomics. (2015) 12:57–65. 25770188

[48] Rodríguez H Levican J Muñoz J Carrillo D Acevedo M Gaggero A . Viral infections in prostate carcinomas in Chilean patients. Infect Agent Cancer. (2015) 10:27. doi: 10.1186/s13027-015-0024-y. PMID: 26330890 PMC4556319

[49] Akgül B Pfister D Knüchel R Heidenreich A Wieland U Pfister H . No evidence for a role of xenotropic murine leukaemia virus-related virus and BK virus in prostate cancer of German patients. Med Microbiol Immunol. (2012) 201:245–8. doi: 10.1007/s00430-011-0215-0. PMID: 21898167

[50] Sfanos K Sauvageot J Fedor H Dick J De Marzo A Isaacs W . A molecular analysis of prokaryotic and viral DNA sequences in prostate tissue from patients with prostate cancer indicates the presence of multiple and diverse microorganisms. Prostate. (2008) 68:306–20. doi: 10.1002/pros.20680. PMID: 18163428

[51] Balis V Sourvinos G Soulitzis N Giannikaki E Sofras F Spandidos DA . Prevalence of BK virus and human papillomavirus in human prostate cancer. Int J Biol Markers. (2007) 22:245–51. doi: 10.1177/172460080702200402. PMID: 18161654

[52] Das D Shah RB Imperiale MJ . Detection and expression of human BK virus sequences in neoplastic prostate tissues. Oncogene. (2004) 23:7031–46. doi: 10.1038/sj.onc.1207920. PMID: 15258563

[53] Zambrano A Kalantari M Simoneau A Jensen JL Villarreal LP . Detection of human polyomaviruses and papillomaviruses in prostatic tissue reveals the prostate as a habitat for multiple viral infections. Prostate. (2002) 53:263–76. doi: 10.1002/pros.10157. PMID: 12430138

[54] Dumoulin A Hirsch HH . Reevaluating and optimizing polyomavirus BK and JC real-time PCR assays to detect rare sequence polymorphisms. J Clin Microbiol. (2011) 49:1382–8. doi: 10.1128/JCM.02008-10 PMC3122792 21325560

[55] Zhong S Randhawa PS Ikegaya H Chen Q Zheng HY Suzuki M . Distribution patterns of BK polyomavirus (BKV) subtypes and subgroups in American, European and Asian populations suggest co-migration of BKV and the human race. J Gen Virol. (2009) 90:144–52. doi: 10.1099/vir.0.83611-0. PMID: 19088283

[56] Stultz J Fong L . How to turn up the heat on the cold immune microenvironment of metastatic prostate cancer. Prostate Cancer Prostatic Dis. (2021) 24:697–717. doi: 10.1038/s41391-021-00340-5. PMID: 33820953 PMC8384622

[57] Kwon JTW Bryant RJ Parkes EE . The tumor microenvironment and immune responses in prostate cancer patients. Endocr Relat Cancer. (2021) 28:T95–T107. doi: 10.1530/ERC-21-0149. PMID: 34128831 PMC8345898

[58] Abend JR Imperiale MJ . Transforming growth factor-beta-mediated regulation of BK virus gene expression. Virology. (2008) 378:6–12. doi: 10.1016/j.virol.2008.05.009. PMID: 18559281 PMC2569840

[59] Li YJ Wang JW Wu HH Wang HH Chiang YJ Yang HY . Tumor necrosis factor-alpha blockade suppresses BK polyomavirus replication. Infection. (2023) 51:967–80. doi: 10.1007/s15010-022-01962-0. PMID: 36512270 PMC9745287

[60] Signorini L Croci M Boldorini R Varella RB Elia F Carluccio S . Interaction between human polyomavirus BK and hypoxia inducible factor-1 alpha. J Cell Physiol. (2016) 231:1343–9. doi: 10.1002/jcp.25238. PMID: 26529465

[61] Kenan DJ Mieczkowski PA Burger-Calderon R Singh HK Nickeleit V . The oncogenic potential of BK-polyomavirus is linked to viral integration into the human genome. J Pathol. (2015) 237:379–89. doi: 10.1002/path.4584. PMID: 26172456 PMC5042064

[62] Wang Y Liu Y Deng W Fu F Yan S Yang H . Viral integration in BK polyomavirus-associated urothelial carcinoma in renal transplant recipients: multistage carcinogenesis revealed by next-generation virome capture sequencing. Oncogene. (2020) 39:5734–42. doi: 10.1038/s41388-020-01398-6. PMID: 32724161

[63] Jin Y Zhou Y Deng W Wang Y Lee RJ Liu Y . Genome-wide profiling of BK polyomavirus integration in bladder cancer of kidney transplant recipients reveals mechanisms of the integration at the nucleotide level. Oncogene. (2021) 40:46–54. doi: 10.1038/s41388-020-01502-w. PMID: 33051598

[64] Wang Y Yan S Liu Y Yan Z Deng W Geng J . Dynamic viral integration patterns actively participate in the progression of BK polyomavirus-associated diseases after renal transplantation. Am J Transplant. (2023) 23:1694–708. doi: 10.1016/j.ajt.2023.07.014. PMID: 37507072

