# Supplementary material for: Association between BK polyomavirus and prostate cancer: a systematic review and meta-analysis
Source: Front Oncol. 2026 May 8;16:1764752. doi: 10.3389/fonc.2026.1764752 (PMC13193935; doi:10.3389/fonc.2026.1764752)
Supplement: Supplementary Figure S1 — Leave-one-out sensitivity analysis for pooled prevalence. Influence analysis showing the pooled prevalence estimate after sequential omission of each individual study. [file DataSheet1.doc]

Supplementary Material

**Supplementary Figure S1**


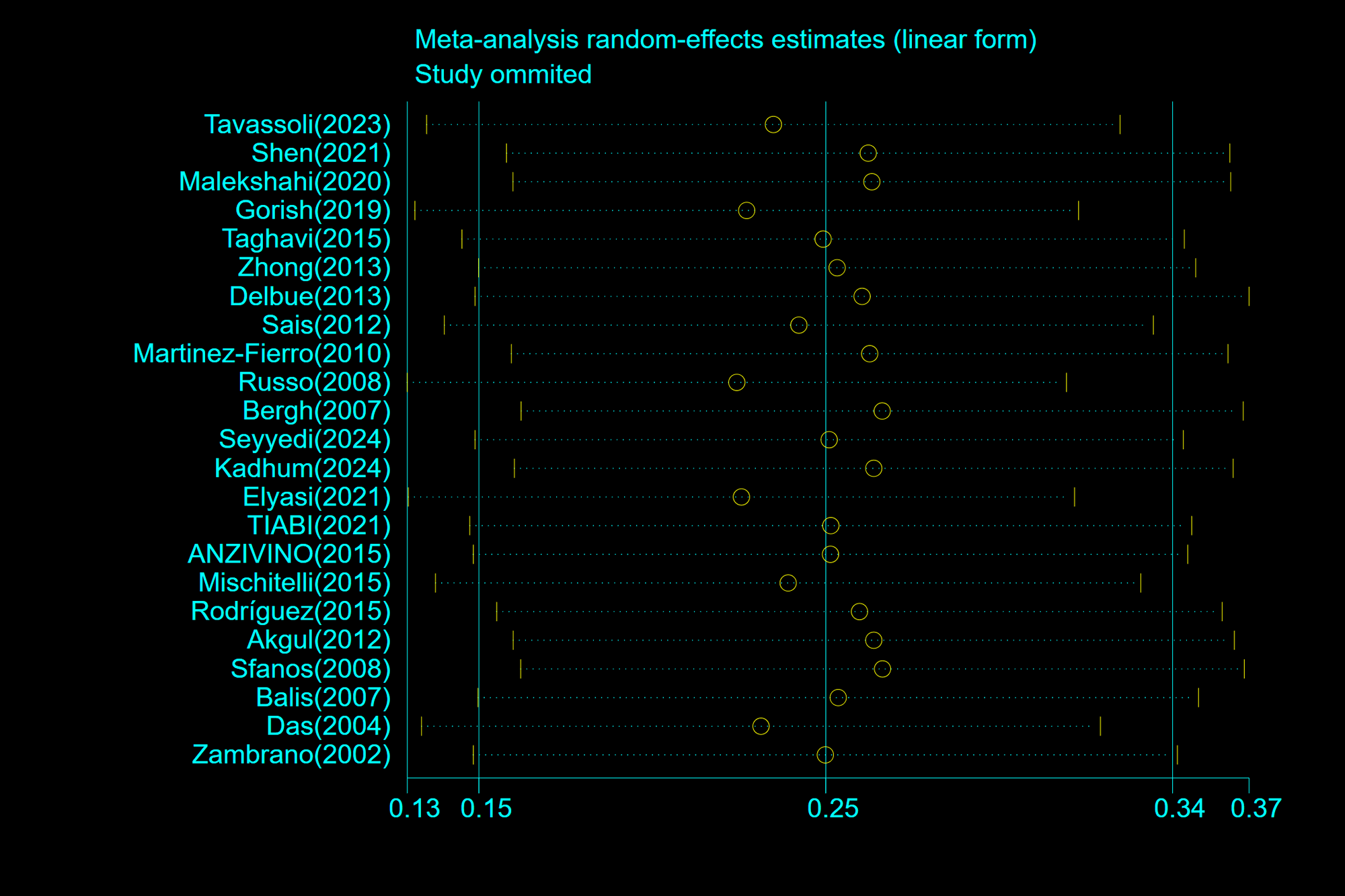


Leave-one-out sensitivity analysis for pooled prevalence. Influence analysis showing the pooled prevalence estimate after sequential omission of each individual study.

**Supplementary Figure S2**


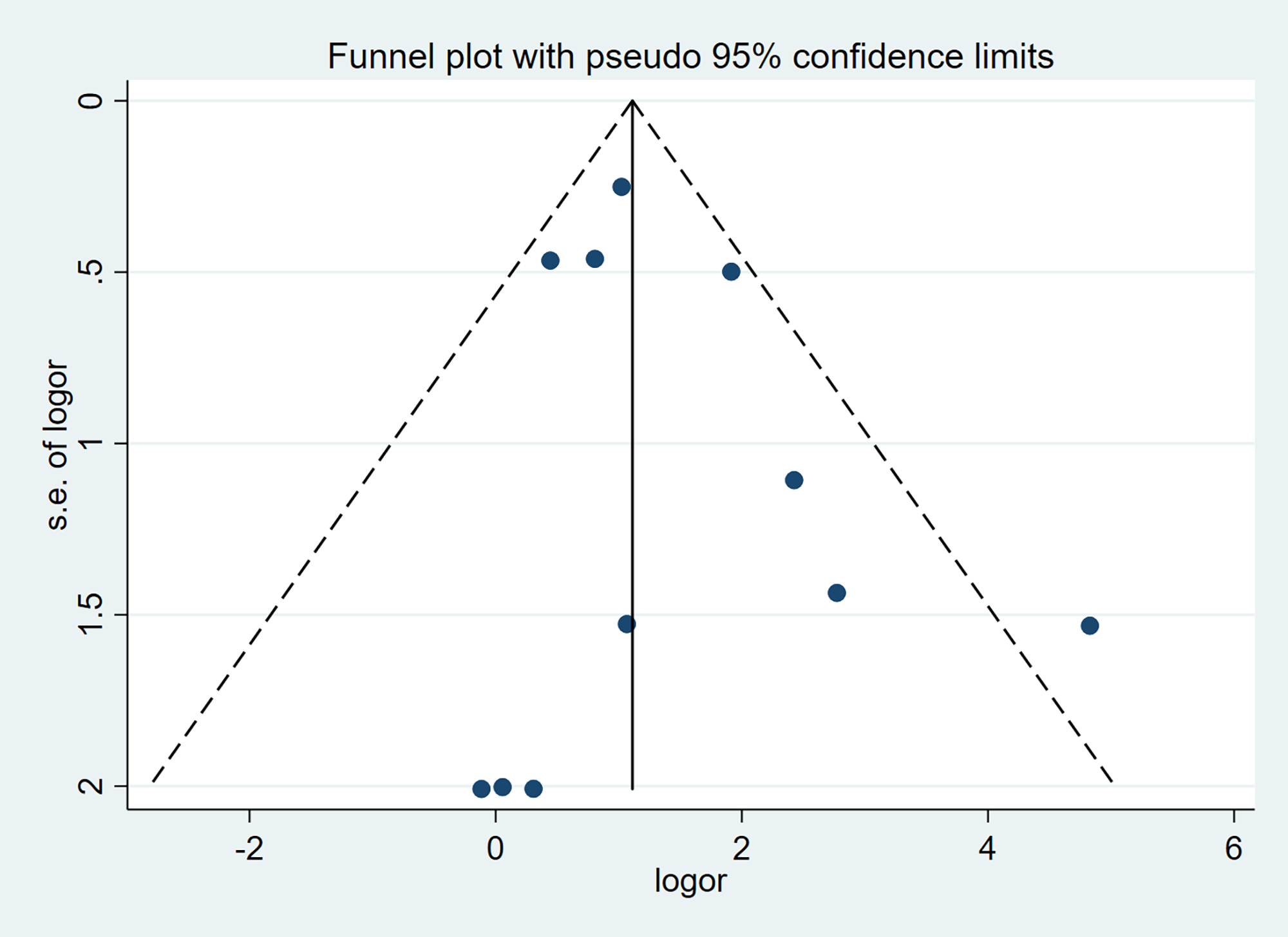


Funnel plot for the meta-analysis of the association between BKPyV infection and PCa.

**Supplementary Figure S3**

**
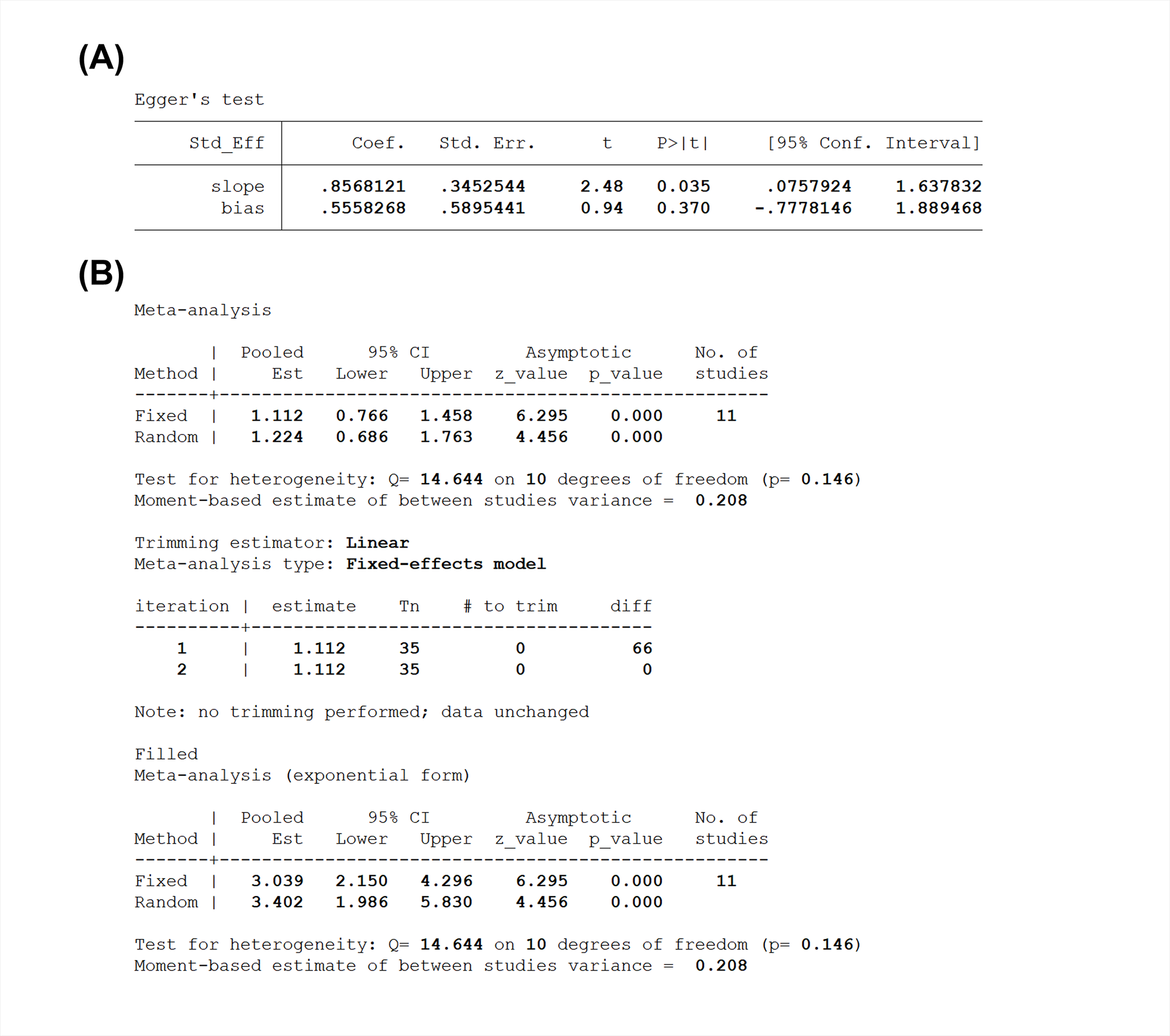
**

Evaluation of publication bias for the association between BK polyomavirus infection and prostate cancer. (**A**) Egger’s test plot; (**B**) Results of the Trim and Fill analysis.

**Supplementary Figure S4**

**
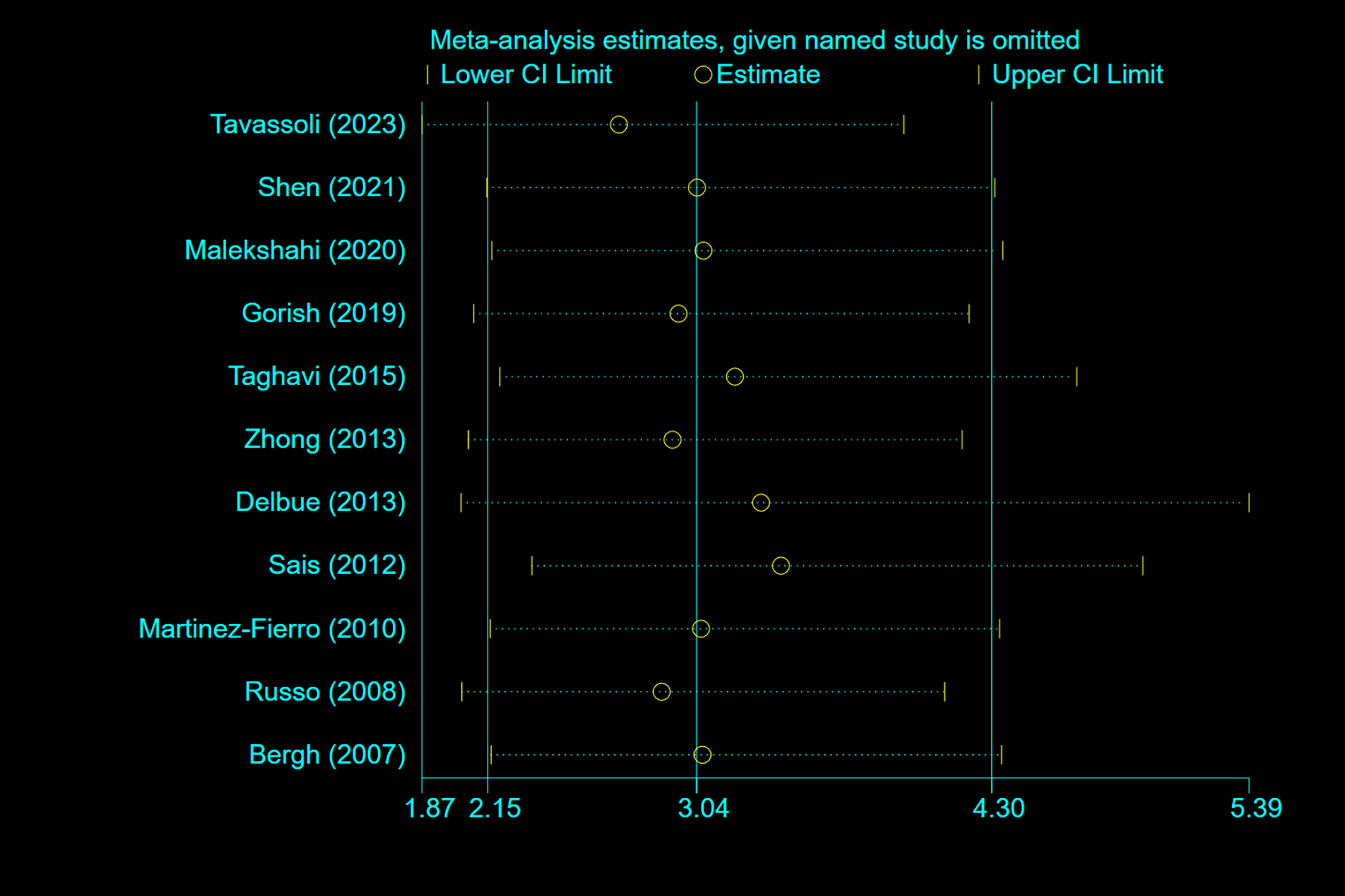
**

Leave-one-out sensitivity analysis for pooled OR. Influence analysis showing the pooled OR estimate after sequential omission of each individual study.

| First author | Year | Country | Study type | Detection method | Sample type | PCR target region | PCa cases (Pos/Total) | Controls (Pos/Total) |
| --- | --- | --- | --- | --- | --- | --- | --- | --- |
| Tavassoli^(23)^ | 2023 | Iran | Case-control | Nested-PCR | FFPE tissue | TAg | 26/49 | 7/49 |
| Shen^(18)^ | 2021 | China | Case-control | Nested-PCR | FFPE tissue | TAg | 3/76 | 0/30 |
| Malekshahi^(24)^ | 2020 | Iran | Case-control | PCR | FFPE tissue | TAg | 0/64 | 0/57 |
| Gorish^(34)^ | 2019 | Sudan | Case-control | PCR | FFPE tissue | TAg | 16/17 | 2/4 |
| Taghavi^(35)^ | 2015 | Iran | Case-control | PCR | FFPE tissue | VP1 | 17/60 | 9/60 |
| Zhong^(36)^ | 2013 | China | Case-control | Nested-PCR and PCR | Fresh tissue | TAg and VP1 | 6/32 | 1/50 |
| Delbue^(37)^ | 2013 | Italy | Case-control | qPCR | Fresh tissue | VP1 | 55/328 | 26/385 |
| Sais^(38)^ | 2012 | Switzerland | Case-control | qPCR | FFPE tissue | TAg | 18/43 | 12/38 |
| Martinez-Fierro^(39)^ | 2010 | Mexico | Case-control | Nested-PCR | Fresh tissue | TAg | 0/55 | 0/75 |
| Russo^(40)^ | 2008 | Italy | Case-control | qPCR | Fresh tissue | TAg | 22/26 | 0/12 |
| Bergh^(25)^ | 2007 | Sweden | Case-control | Nested-PCR | FFPE tissue | VP1 | 0/171 | 0/181 |
| Seyyedi^(41)^ | 2024 | Iran | Cross-sectional | Semi-Nested PCR | FFPE tissue | TAg | 3/13 |  |
| Kadhum^(42)^ | 2024 | Iraq | Cross-sectional | qPCR | FFPE tissue | TAg | 0/74 |  |
| Elyasi^(43)^ | 2021 | Iran | Cross-sectional | Semi-Nested PCR | FFPE tissue | TAg | 33/50 |  |
| TIABI^(44)^ | 2021 | Morocco | Cross-sectional | PCR | Fresh tissue | Not reported | 12/50 |  |
| ANZIVINO^(45)^ | 2015 | Italy | Cross-sectional | qPCR | Fresh tissue | TAg | 6/26 |  |
| Mischitelli^(46)^ | 2015 | Italy | Cross-sectional | qPCR | Fresh tissue | Not reported | 31/71 |  |
| Rodríguez^(47)^ | 2015 | Chile | Cross-sectional | qPCR | FFPE tissue | TAg | 6/69 |  |
| Akgul^(48)^ | 2012 | Germany | Cross-sectional | qPCR | FFPE tissue | Not reported | 1/85 |  |
| Sfanos^(49)^ | 2008 | USA | Cross-sectional | Nested-PCR | Fresh tissue | TAg | 1/200 |  |
| Balis^(50)^ | 2007 | Greece | Cross-sectional | PCR | FFPE tissue and fresh tissue | VP1 | 8/42 |  |
| Das^(28)^ | 2004 | USA | Cross-sectional | PCR | FFPE tissue | TAg | 16/21 |  |
| Zambrano^(51)^ | 2002 | USA | Cross-sectional | Nested-PCR | FFPE tissue and fresh tissue | TAg | 2/7 |  |

**Table 1.** Characteristics of studies included in the systematic review and meta-analysis.

| **Search** | **Query** |
| --- | --- |
| #1 | ((Prostatic Neoplasms) OR (Neoplasms, Prostatic) OR (Neoplasm, Prostatic) OR (Prostatic Neoplasm) OR (Prostate Neoplasms) OR (Neoplasms, Prostate) OR (Neoplasm, Prostate ) OR (Prostate Neoplasm) OR (Prostate Cancer) OR (Cancer, Prostate) OR (Cancers, Prostate ) OR (Prostate Cancers) OR (Cancer of Prostate) OR (Cancer of the Prostate) OR (Prostatic Cancer) OR (Cancer, Prostatic) OR (Cancers, Prostatic) OR (Prostatic Cancers)) |
| #2 | ((BK Virus) OR (Human Polyomavirus BK ) OR (Polyomavirus BK, Human ) OR (BK polyomavirus ) OR (Polyomavirus hominis 1 ) OR (Polyomavirus, BK )) |
| #3 | ((Polyomavirus) OR (Polyomaviruses ) OR (Polyoma Virus ) OR (Viruses, Polyoma ) OR (Virus, Polyoma ) OR (Polyoma Viruses ) OR (Bovine polyomavirus ) OR (Bovine polyomaviruses ) OR (polyomaviruses, Bovine ) OR (Hamster polyomavirus ) OR (Hamster polyomaviruses ) OR (polyomavirus, Hamster ) OR (Murine polyomavirus ) OR (Murine polyomaviruses ) OR (polyomaviruses, Murine )) |
| #4 | #1 AND (#2 OR #3)  ((Prostatic Neoplasms) OR (Neoplasms, Prostatic) OR (Neoplasm, Prostatic) OR (Prostatic Neoplasm) OR (Prostate Neoplasms) OR (Neoplasms, Prostate) OR (Neoplasm, Prostate ) OR (Prostate Neoplasm) OR (Prostate Cancer) OR (Cancer, Prostate) OR (Cancers, Prostate ) OR (Prostate Cancers) OR (Cancer of Prostate) OR (Cancer of the Prostate) OR (Prostatic Cancer) OR (Cancer, Prostatic) OR (Cancers, Prostatic) OR (Prostatic Cancers)) AND (((BK Virus) OR (Human Polyomavirus BK ) OR (Polyomavirus BK, Human ) OR (BK polyomavirus ) OR (Polyomavirus hominis 1 ) OR (Polyomavirus, BK )  ) OR ((Polyomavirus) OR (Polyomaviruses ) OR (Polyoma Virus ) OR (Viruses, Polyoma ) OR (Virus, Polyoma ) OR (Polyoma Viruses ) OR (Bovine polyomavirus ) OR (Bovine polyomaviruses ) OR (polyomaviruses, Bovine ) OR (Hamster polyomavirus ) OR (Hamster polyomaviruses ) OR (polyomavirus, Hamster ) OR (Murine polyomavirus ) OR (Murine polyomaviruses ) OR (polyomaviruses, Murine ))) |

**Supplementary Table S1.** Search strategy

**Supplementary Table S2.** Newcastle-Ottawa Scale for cross-sectional studies

| **Categories and items** | **Tool for cross-sectional studies** |
| --- | --- |
| **Selection: (Maximum 5 stars)** | |
| Representativeness of the sample | 1. Truly representative of the average in the target population. * (* = 1 point) (all subjects or random sampling) b) Somewhat representative of the average in the target population. * (non-random sampling)   c)Selected group of users.  d) No description of the sampling strategy. |
| Sample size | 1. Justified and satisfactory. * 2. Not justified. |
| Non-respondents | 1. Comparability between respondents and non-respondents characteristics is established, and the response rate is satisfactory. * 2. The response rate is unsatisfactory, or the comparability between respondents and non-respondents is unsatisfactory.   c) No description of the response rate or the characteristics of the responders and the non-responders. |
| Ascertainment of the exposure (risk factor) | 1. Validated measurement tool. ** (** = 2 point) 2. Non-validated measurement tool, but the tool is available or described.*   c) No description of the measurement tool. |
| **Comparability: (Maximum 2 stars)** | |
| The subjects in different outcome groups are comparable, based on the study design or analysis. Confounding factors are controlled. | 1. The study controls for the most important factor (select one). *   b) The study control for any additional factor. * |
| **Outcome: (Maximum 3 stars)** | |
| Assessment of the outcome | 1. Independent blind assessment. ** 2. Unblinded assessment. ** 3. Used non-standard. *   d) No description. |
| Statistical test | 1. The statistical test used to analyze the data is clearly described and appropriate, and the measurement of the association is presented, including confidence intervals and the probability level (*p* value). *   b) The statistical test is not appropriate, not described or incomplete |

**Supplementary Table S3. NOS Checklist for selected studies (case-control study)**

|  | Selection |  |  |  | Comparability | Exposure |  |  | Score |
| --- | --- | --- | --- | --- | --- | --- | --- | --- | --- |
| Reference | Is the case  definition  adequate?  (1) | Represen-  tativeness of the cases  (1) | Selection of  Controls  (1) | Definition of  Controls  (1) | Comparability of cases and controls on the basis of the Design or analysis  (2) | Ascertainment of exposure  (2) | Same method  of ascertain-  ment for cases  and controls(1) | Non-Response  Rate  (1) | Total |
| Tavassoli,2023,Iran ^(27)^ | 1 | 0 | 0 | 0 | 1 | 1 | 1 | 1 | 5 |
| Shen,2021,China ^(19)^ | 1 | 0 | 0 | 1 | 1 | 1 | 1 | 1 | 6 |
| Malekshahi,2020,Iran ^(28)^ | 1 | 0 | 0 | 0 | 1 | 1 | 1 | 1 | 5 |
| Gorish,2019,Sudan ^(35)^ | 1 | 0 | 0 | 1 | 2 | 1 | 1 | 1 | 7 |
| Taghavi,2015,Iran ^(36)^ | 1 | 0 | 0 | 0 | 1 | 1 | 1 | 1 | 5 |
| Zhong,2013,China ^(37)^ | 1 | 0 | 0 | 1 | 1 | 1 | 1 | 1 | 6 |
| Delbue,2013,Italy ^(38)^ | 1 | 0 | 0 | 1 | 1 | 1 | 1 | 1 | 6 |
| Sais,2012,Switzerland ^(39)^ | 1 | 0 | 0 | 1 | 1 | 1 | 1 | 1 | 6 |
| Martinez-Fierro,2010,Mexico ^(40)^ | 1 | 0 | 0 | 1 | 1 | 1 | 1 | 1 | 6 |
| Russo,2009,Italy ^(41)^ | 1 | 0 | 0 | 1 | 1 | 1 | 1 | 1 | 6 |
| Bergh,2007,Sweden ^(29)^ | 1 | 0 | 1 | 1 | 1 | 1 | 1 | 1 | 7 |

NOS = Newcastle–Ottawa Scale

**Supplementary Table S4. NOS Checklist for selected studies (cross-sectional study)**

|  | Selection |  |  |  | Comparability | Outcome |  | Score |
| --- | --- | --- | --- | --- | --- | --- | --- | --- |
| Reference | Representativeness of the sample  (1) | Sample size  (1) | Non-respondents  (1) | Ascertainment of the exposure (risk factor) (2) | The subjects in different outcome groups are comparable, based on the study design or analysis. Confounding factors are controlled  (2) | Assessment of the outcome  (2) | Statistical test (1) | Total |
| Seyyedi,2024,Iran ^(42)^ | 1 | 0 | 0 | 2 | 1 | 2 | 1 | 7 |
| Kadhum,2024,Iraq ^(43)^ | 1 | 0 | 0 | 2 | 0 | 2 | 1 | 6 |
| Elyasi,2021,Iran ^(44)^ | 1 | 0 | 0 | 2 | 1 | 2 | 1 | 7 |
| TIABI,2021,Morocco ^(45)^ | 1 | 0 | 0 | 2 | 2 | 2 | 1 | 8 |
| ANZIVINO,2015,Italy ^(46)^ | 1 | 0 | 0 | 2 | 0 | 2 | 1 | 6 |
| Mischitelli,2015,Italy ^(47)^ | 1 | 0 | 0 | 2 | 1 | 2 | 1 | 7 |
| Rodríguez,2015,Chile ^(48)^ | 1 | 0 | 0 | 2 | 2 | 2 | 1 | 8 |
| Akgul,2012,Germany ^(49)^ | 1 | 0 | 0 | 2 | 2 | 2 | 1 | 8 |
| Sfanos,2008,USA ^(50)^ | 1 | 0 | 0 | 2 | 1 | 2 | 0 | 6 |
| Balis,2007,Greece ^(51)^ | 1 | 0 | 0 | 2 | 1 | 2 | 1 | 7 |
| Das,2004,USA ^(52)^ | 1 | 0 | 0 | 2 | 0 | 2 | 0 | 5 |
| Zambrano,2002,USA ^(53)^ | 1 | 0 | 0 | 2 | 1 | 2 | 0 | 6 |

**Supplementary Table S5.** Heterogeneity statistics for meta-analyses of BKPyV prevalence in PCa tissues

| Characteristics | Categories | No. of studies | Pooled prevalence (%) (95% CI) | Q (df), I^2^, P-value | Test for heterogeneity between sub-groups (Qb (df) , P-value) |
| --- | --- | --- | --- | --- | --- |
| Overall |  | 23 | 22% (12-34%) | 589.97 (22), 96.27%, p<0.01 |  |
| Detection method | Nested-/Semi-nested PCR | 8 | 14% (1-35%) | 227.68 (7), 96.93%, p<0.01 | 1.53 (2), p=0.47 |
|  | PCR | 6 | 36% (10-67%) | 119.40 (5), 95.81%, p<0.01 |  |
|  | qPCR | 8 | 22% (8-40%) | 169.02 (7), 95.86%, p<0.01 |  |
| Specimen type | FFPE tissue | 13 | 23% (8-42%) | 387.36 (12), 96.90%, p<0.01 | 0.02 (1), p=0.89 |
|  | Fresh tissue | 8 | 21% (7-40%) | 196.88 (7), 96.44%, p<0.01 |  |
| PCR target region | TAg | 15 | 27% (10-47%) | 439.79 (14), 96.82%, p<0.01 | 1.30 (1), p=0.25 |
|  | VP1 | 4 | 13% (1-32%) | 86.14 (3), 96.52%, p<0.01 |  |
| Study location | Asia | 8 | 18% (3-41%) | 176.02 (7), 96.04%, p<0.01 | 0.24 (2), p=0.88 |
|  | Europe | 8 | 23% (8-44%) | 221.87 (7), 96.85%, p<0.01 |  |
|  | North America/South America/Africa | 7 | 26% (5-55%) | 176.00 (6), 96.59%, p<0.01 |  |
| Gleason score | 6 | 14 | 20% (8-35%) | 32.85 (13), 60.42%, p<0.01 |  |
|  | 7 | 14 | 28% (13-46%) | 86.62 (13), 84.99%, p<0.01 |  |
|  | 8-10 | 14 | 41% (13-71%) | 143.01 (13), 91.61%, p<0.01 |  |

**Supplementary Table S6.** Heterogeneity statistics for meta-analyses of the association between BKPyV positivity and PCa

| Characteristics | Categories | No. of studies | Pooled OR (95% CI) | Q (df), I^2^, P-value | Test for heterogeneity between sub-groups (Qb (df) , P-value) |
| --- | --- | --- | --- | --- | --- |
| Overall |  | 11 | 3.04 (2.15-4.30) | 14.64 (10), 31.7%, p=0.146 |  |
| Detection method | Nested PCR | 4 | 5.29 (2.19-12.78) | 1.51 (3), 0.0%, p=0.681 | 2.05 (2), p=0.359 |
|  | PCR | 3 | 2.56 (1.10-5.94) | 1.99 (2), 0.0%, p=0.370 |  |
|  | qPCR | 3 | 2.65 (1.72-4.06) | 7.66 (2), 73.9%, p=0.022 |  |
| Specimen type | FFPE tissue | 7 | 2.83 (1.70-4.71) | 6.99 (6), 14.2%, p=0.322 | 0.14 (1), p=0.709 |
|  | Fresh tissue | 4 | 3.23 (2.02-5.17) | 7.52 (3), 60.1%, p=0.057 |  |
| PCR target region | TAg | 7 | 3.69 (2.01-6.76) | 12.00 (6), 50.0%, p=0.062 | 0.82 (1), p=0.366 |
|  | VP1 | 3 | 2.62 (1.70-4.02) | 0.38 (2), 0.0%, p=0.828 |  |
| Study location | Asia | 5 | 3.90 (2.11-7.20) | 4.18 (4), 4.3%, p=0.382 | 1.60 (2), p=0.448 |
|  | Europe | 4 | 2.62 (1.71-4.01) | 7.86 (3), 61.9%, p=0.049 |  |
|  | North America/Africa | 2 | 6.95 (0.70-68.56) | 1.00 (1), 0.0%, p=0.318 |  |
| Gleason score | 6 | 8 | 6.43 (3.69-11.21) | 2.39 (7), 0.0%, p=0.935 |  |
|  | 7 | 8 | 3.27 (1.90-5.65) | 11.33 (7), 38.2%, p=0.125 |  |
|  | 8-10 | 8 | 4.46 (2.26-8.79) | 15.81 (7), 55.7%, p=0.027 |  |
